# Bi-mediated allylation of aldehydes in [bmim][Br]: a mechanistic investigation

**DOI:** 10.3762/bjoc.14.193

**Published:** 2018-08-22

**Authors:** Mrunesh Koli, Sucheta Chatterjee, Subrata Chattopadhyay, Dibakar Goswami

**Affiliations:** 1 Bio-Organic Division, Bhabha Atomic Research Centre, Mumbai - 400 085, India; 2 Homi Bhabha National Institute, Training School Complex, Anushakti Nagar, Mumbai - 400 094, India

**Keywords:** allylation, bismuth, [bmim][Br], ionic liquid

## Abstract

The inexpensive room temperature ionic liquid (RTIL), [bmim][Br] has been found to be a superior medium for the Bi-mediated Barbier-type allylation of aldehydes compared to other conventional solvents. It plays the dual role of a solvent and a metal activator enabling higher yields of the products in a shorter reaction time using stoichiometric/near-stoichiometric amounts of reagents. Plausibly, [bmim][Br] activates Bi metal by a charge transfer mechanism. The ^1^H VT-NMR studies suggested that both the allylating species, allylbismuth dibromide and diallylbismuth bromide, are generated in situ.

## Introduction

The metal-mediated Barbier-type allylation of aldehydes has drawn considerable attention, because the resultant homoallylic alcohols are versatile intermediates for natural product synthesis [1–7]. The reaction, carried out in organic solvents, water, mixed solvent systems and room temperature ionic liquids (RTILs) is also ideal for probing in situ formation of different allylmetal species in solution, their stability and reactivity towards electrophiles [8–10]. Despite extensive investigation, several key factors of the reaction have not been adequately addressed. In modern era, the development of green chemical routes has become inevitable for sustainable technologies. To this end, RTILs are conceived as eco-friendly solvents due to their low vapor pressure, high stability towards air, moisture and heat, ability to dissolve various substrates, and their reusability [11–14]. However, issues such as use of large excess of the reagents, solvents, metals and toxic metal activators such as acids or fluorides are the major limitations of the reported protocols of this reaction [15–18]. Other methods such as Rieke’s activation [19], metal-graphite [20] etc. are also tedious and not ideal for green chemistry. The use of a second metal with lower reduction potential than the active metals could not reduce the amounts of disposable metallic wastes [21–22]. Since most of the in situ-generated allylmetals are hydrolytically unstable, a large excess of reagents is used for carrying out the reaction in water [23–24].

Although several metals have been used for the reaction, those with Zn, In and Sn are more widely investigated [1,25]. However, Bi is cheaper, less toxic [26], and has more metallic character [27]. Previously, the Bi-mediated Barbier-type allylations of carbonyls have been reported in organic solvents [28–30], water [31] or under solvent-free conditions [32–33]. The solvent free synthetic procedure required a large excess (4–8 equiv) of Bi metal [33] whereas, the reactions in water or in organic solvents required either aqueous KF [31] or aqueous HBr [34] as the metal activator. As an alternative, Xu et al. found nano-Bi to be more effective than regular Bi-powder, although this method had an intrinsic difficulty of preparing Bi-nanoparticles via reduction of Bi(III) salts [35]. These apart, combinations of Bi(III) salts with reducing metals, e.g., Mg-BiCl_3_ [36], Fe-BiCl_3_ or Zn-BiCl_3_ [30], and Al-BiCl_3_ [37] have been used. Aqueous NH_4_Cl was also employed as an additive in the Al/BiCl_3_ mediated allylation of carbonyls in aqueous THF [38].

## Results and Discussion

**Scheme 1:**
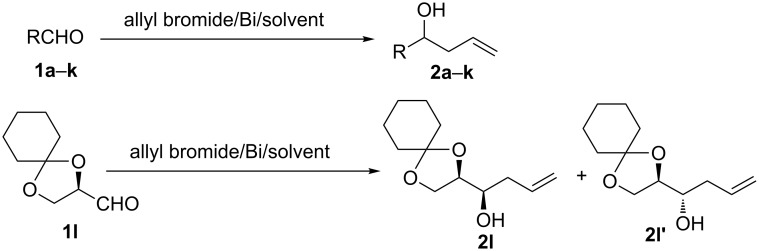
Bi-mediated allylation of aldehydes.

Initially, we screened different solvents and metal activators (chemical additives and ultrasonication) for the Bi-mediated allylation of benzaldehyde (**1a**), as the model substrate with commercially available and inexpensive allyl bromide (Scheme 1) at room temperature (25 °C) [39–42]. The results are shown in Table 1. As reported earlier [30], the reaction carried out in DMF took a long time for completion and gave the product **2a** with a moderate yield (Table 1, entry 1). Increasing the amounts of Bi and allyl bromide did not improve the reaction outcome (Table 1, entry 2). Allylations in other organic solvents such as MeCN and THF were inferior, and furnished **2a** in lesser yields (Table 1, entries 3–8) even under metal activation by KF (Table 1, entries 5 and 8) or ultrasonication (Table 1, entries 4 and 7). Reduction in the amounts of Bi and allyl bromide under these conditions led to significantly poorer results (data not shown). The use of the mixed solvent THF/H_2_O (1:1, v/v) at room temperature or under sonication increased the reaction time (14 h), but furnished **2a** in similar yields (60–65%) as obtained in DMF or MeCN-KF (Table 1, entries 9 and 10 vis-à-vis 2 and 4). In water, the reaction yield was modest (entry 11). Ultrasonication in water gave a similar result (entry 12), but activation with aqueous KF boosted the yield to 72% and reduced the reaction time (3 h, Table 1, entry 13). The reaction in [bmim][PF_6_] was sluggish and furnished **2a** in 41% yield after 14 h (Table 1, entry 14), but the commonly used RTIL, [bmim][BF_4_] was totally ineffective (Table 1, entry 15). True to our expectation, the reaction was very fast in [bmim][Br] and furnished **2a** in 88% yield. Notably, the reaction proceeded to completion with almost stoichiometric amounts of allyl bromide (1.2 equiv) and Bi (1.0 equiv) in absence of any additional metal activator (Table 1, entry 16). In addition, as previously reported in case of crotylation [40], the allylation did not proceed in absence of oxygen (Table 1, entry 17), or when the C-2 imidazole proton was absent in the RTIL (in 1-butyl-2,3-dimethylimidazolium bromide ([bmmim][Br]), Table 1, entry 18). This result is significant, since, to the best of our knowledge, this is the first report of Bi-mediated allylation of aldehydes in an RTIL.

**Table 1:** Effect of the reaction conditions on the Bi-mediated allylation of **1a**^a^.

entry	allyl bromide (equiv)	Bi (equiv)	solvent	additive	time (h)	yield of **2a**^b^

1	1.5	1.5	DMF	–	12	65
2	2.5	2.0	DMF	–	12	67
3	2.5	2.0	MeCN	–	12	52
4	2.5	2.0	MeCN	–	10^d^	55
5	2.5	2.0	MeCN	KF^c^	12	62
6	2.5	2.0	THF	–	10	56
7	2.5	2.0	THF	–	12^d^	58
8	2.5	2.0	THF	KF^c^	12	40
9	2.5	2.0	THF:H_2_O^e^	–	14	60
10	2.5	2.0	THF:H_2_O^e^	–	14^d^	65
11	2.5	2.0	H_2_O	–	11	45
12	2.5	2.0	H_2_O	–	10^d^	48
13	2.5	2.0	H_2_O	KF^c^	3	72
14	2.5	2.0	[bmim][PF_6_]	–	14	41
15	2.5	2.0	[bmim][BF_4_]	–	NR^f^	NR^f^
16	1.2	1.0	[bmim][Br]	–	3	88
17	1.2	1.0	[bmim][Br]^h^	–	12	NR^f^
18	1.2	1.0	[bmmim][Br]^g^	–	12	NR^f^

^a^The reactions were carried out at 3 mmol scale as detailed in the experimental section. ^b^Isolated yield. ^c^15 mmol KF was used. ^d^Under ultrasonic irradiation. ^e^THF/H_2_O (1:1, v/v) was used. ^f^NR: No reaction. ^g^1-Butyl-2,3-dimethylimidazolium bromide is abbreviated as [bmmim][Br]. ^h^[bmim][Br] was used after overnight purging with N_2_(g).

Taken together, the above data revealed that [bmim][Br] may be best suited for the Bi-mediated allylation. To probe the generality of the methodology, several aromatic and aliphatic aldehydes **1b**–**l** were subjected to Bi-mediated allylation in [bmim][Br] (Scheme 1, Table 2). The reactions with aromatic aldehydes, possessing both electron-withdrawing (**1b**–**e**) and electron-releasing (**1f**–**h**) substituents were complete within 3–6 h to furnish **2b**–**h** in appreciable yields (Table 2, entries 1–7). Steric hindrance was not detrimental to the yield of the reaction, as it was evident from the allylation of **1e**, bearing a substituent *ortho* to the aldehyde function (Table 2, entry 4). The aliphatic aldehydes **1i** and **1j** also reacted similarly to give the homoallylic alcohols **2i** and **2j**, respectively, in >86% yields (Table 2, entries 8 and 9). Allylation of the conjugated aldehyde **1k** furnished the 1,2-addition product **2k** exclusively, establishing chemoselectivity of the protocol (Table 2, entry 10). With the chiral substrate (*R*)-2,3-*O*-cyclohexylideneglyceraldehyde (**1l**), the *anti*-homoallylic alcohol **2l'** was obtained as the major diastereomer (Table 2, entry 11), although the reaction diastereoselectivity was inferior to that by the Luche’s protocol using Zn metal [42]. In all the reactions, the products were easily isolated by extracting the reaction mixture three times with Et_2_O followed by concentration in vacuo. The reactions were clean without any side-products and unreacted starting materials. We have reused [bmim][Br] three times after discarding the metallic product, BiOBr, settled at the bottom of the flask, without any significant effect (88–85%) on the reaction yields.

**Table 2:** Bi-mediated allylation of different aldehydes in [bmim][Br]^a^.

entry	substrate	R	allyl bromide (equiv)	Bi (equiv)	time (h)	product	yield^b^ (%)

1	**1b**	*p*-Br-C_6_H_4_	1.2	1.0	6	**2b**	86
2	**1c**	*p*-NO_2_-C_6_H_4_	1.2	1.0	3	**2c**	84
3	**1d**	*m*-NO_2_-C_6_H_4_	1.2	1.0	3	**2d**	83
4	**1e**	*o*-Cl-C_6_H_4_	1.2	1.0	3	**2e**	89
5	**1f**	*p*-MeO-C_6_H_4_	1.2	1.0	6	**2f**	87
6	**1g**	*m*-MeO-C_6_H_4_	1.2	1.0	6	**2g**	85
7	**1h**	3,5-(MeO)_2_-C_6_H_3_	1.2	1.0	3	**2h**	91
8	**1i**	C_6_H_13_	1.2	1.0	6	**2i**	86
9	**1j**	C_5_H_11_	1.2	1.0	3	**2j**	90
10	**1k**	C_6_H_5_CH=CH	1.2	1.0	3	**2k**	81
11	**1l**	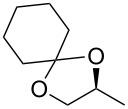	1.2	1.0	3	**2l** + **2l'**	84 (*syn*/*anti* = 32:68)^c^

^a^The reactions were carried out at 3 mmol scale using the same conditions as mentioned in Table 1, entry 16. ^b^Isolated yields of the products. ^c^Diastereomeric ratio determined is based on isolated yields of individual diastereomers.

Overall, the above results clearly established that allylation of a broad spectrum of aldehydes could be realized with Bi in [bmim][Br] in high yields and short reaction times without any additional Bi-activator. The RTIL [bmim][Br] acted both as a solvent and a metal activator, conferring unprecedented advantages in the reaction. In other RTILs, the reaction was either not proceeding at all, or was very sluggish. The specific advantages provided by [bmim][Br] prompted us for further mechanistic studies as discussed below.

### Mechanistic studies

**Figure 1:**
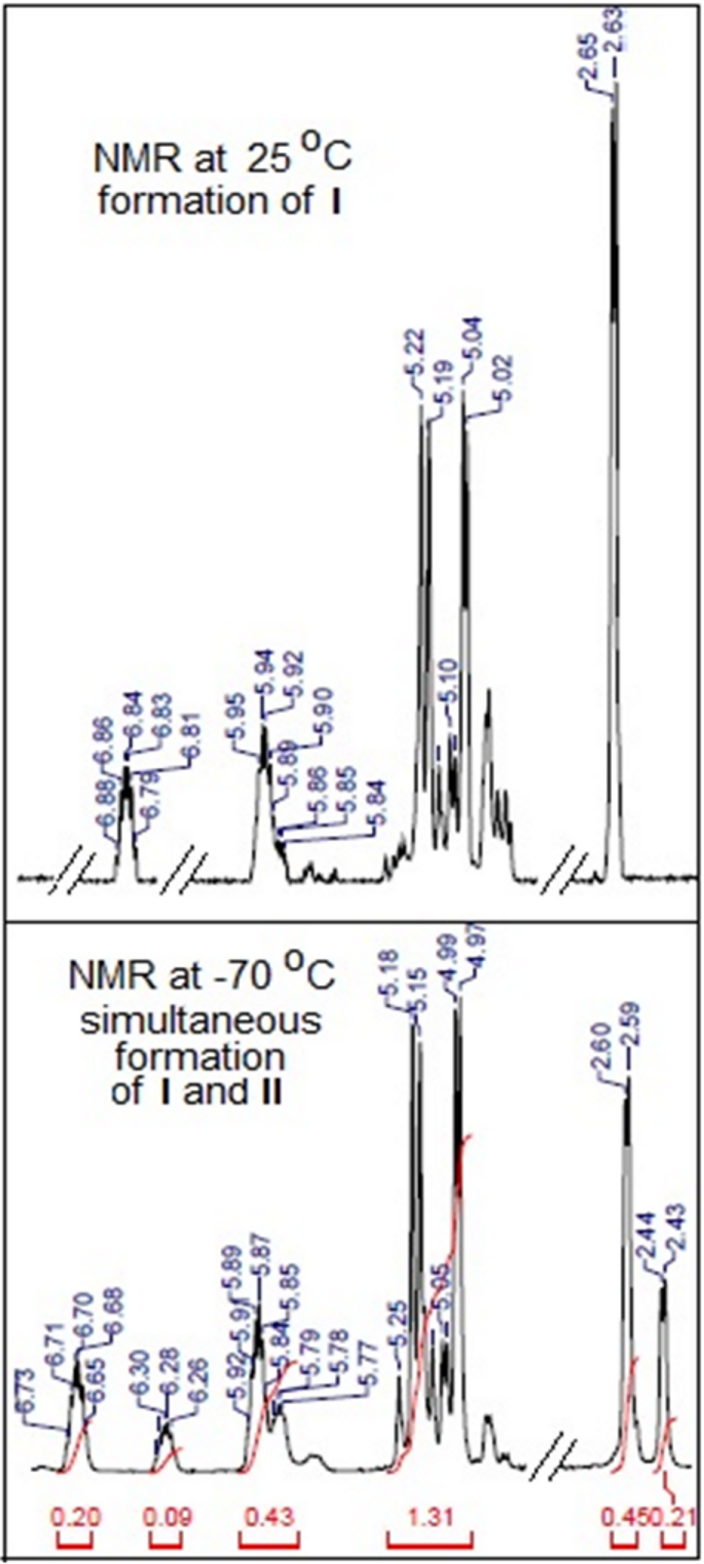
Partial ^1^H NMR spectra (recorded at two temperatures) of the reaction mixture of allyl bromide and Bi stirred in [bmim][Br] for 1 h.

**Figure 2:**
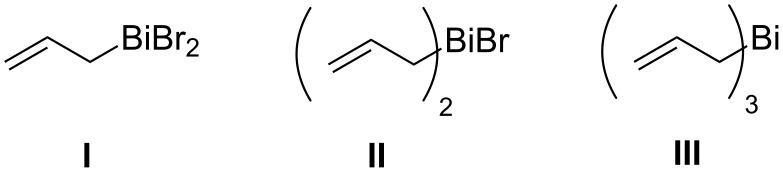
Structures of all the possible allylbismuth species.

For this, we first probed the nature of the organometallic species responsible for the reaction using in situ ^1^H NMR spectroscopy of the reaction mixture comprising of Bi metal (1 mmol) and allyl bromide (1.2 mmol) in [bmim][Br]. After stirring the mixture for 1 h at 25 °C, an aliquot was drawn. Its ^1^H NMR spectrum, recorded in CD_2_Cl_2_ (Figure 1) showed a doublet at δ 2.64 ppm, along with new olefinic multiplets at δ 6.82 ppm. When the ^1^H NMR spectrum of the same aliquot was recorded at –70 °C, two doublets at δ 2.44 and δ 2.60 ppm appeared in 1:2 ratio (taking into account only the integral values in the ^1^H NMR spectrum), along with new olefinic multiplets at δ 6.28 and 6.70 ppm. Among the possible allylbismuth intermediates **I**–**III** (Figure 2), we excluded the possibility of formation of tris(allyl)bismuth (**III**), as the reported [38] doublet at δ 2.33 ppm due to its allylic protons was absent in the ^1^H NMR spectrum of the reaction mixture. Earlier, Jadhav et al. reported [38] formation of only species **I** in water, and characterized it from the allylic proton signals at δ 2.53 ppm in its ^1^H NMR spectrum recorded at an ambient temperature. More recently, Lichtenberg et al. [43] have shown that the ^1^H NMR doublets (δ 2.45 ppm) due to the allylic protons of species **II** can be observed only by recording the ^1^H NMR spectrum at a lower temperature (−95 °C), due to its fluxional behaviour at ambient temperature. These reports prompted us to infer the formation of both allylbismuth dibromide (**I**) and diallylbismuth bromide (**II**) in [bmim][Br], where we could also characterize species **II** only by recording the NMR spectrum at −70 °C. The relative ratio of the integration originated from the allylic signals in ^1^H NMR was 2:1 (Figure 1). However, since species **I** has only one allylic group compared to two in species **II**, stoichiometrically, species **I** and **II** are formed in 4:1 ratio. Nonetheless, the ^1^H NMR spectral pattern clearly indicated formation of η^1^-Bi-allyl coordination complexes. To the best of our knowledge, simultaneous formation of two allylbismuth species in any media is unprecedented.

To see the reactivity of the species **I** and **II**, benzaldehyde (**1a**) was added at −70 °C to the stirred mixture of Bi and allyl bromide in [bmim][Br], and the reaction was followed by ^1^H NMR spectroscopy. However, the peaks corresponding to species **I** and **II**, observed at −70 °C, did not disappear immediately. Probably, benzaldehyde (**1a**) did not react at such a low temperature. Additionally, when the temperature was increased stepwise from −70 °C to 0 °C, we did not observe any peak due to the formation of **2a**. This indicated the inertness of both the allylating species at zero or sub-zero temperature. However, when the mixture was brought to room temperature and stirred for further 30 min, both **I** and **II** disappeared, along with the appearance of a triplet at δ 4.74 ppm, indicating the formation of **2a**. Similar result was also obtained when benzaldehyde (**1a**) was added to the mixture at room temperature. These results clearly indicated that both **I** and **II** act as the active allylating species. However, at this point of time, we are unable to prove the relative reactivities of species **I** and **II** towards **1a** in [bmim][Br]. In analogy to the report [38] describing **I** as the most active allylating species in water medium, we could only presume that **I** is also the most active species for the present protocol. Earlier, we could carry out the Ga-mediated allylation of aldehydes/ketones using a substoichiometric amount of Ga metal, due to the formation of diallyl-GaBr as the only active allylating species [35]. Given that one mole of **II** is expected to react with two moles of the aldehydes, the possibility of substoichiometric amount of Bi metal was explored using **1a** as the substrate. However, the reaction was incomplete (data not shown), and required 1.0 equiv of Bi metal for completion.

**Scheme 2:**
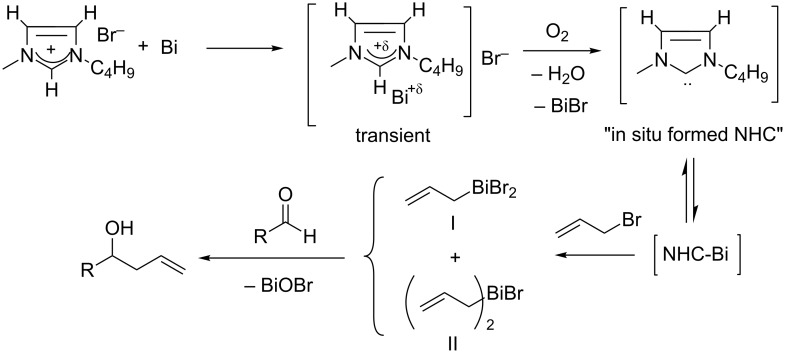
Probable reaction mechanism.

As reported previously [40] in situ activation of Bi metal by [bmim][Br] leading to the generation of an NHC along with BiBr (Scheme 2), was instrumental for the acceleration of the reaction. Eventually, an unstable NHC-Bi complex was formed, which, in presence of allyl bromide, produced both species **I** and **II**. It was also noticed that these reactions do not proceed either in a non-acidic RTIL viz. [bmim][BF_4_], or in absence of oxygen, or in [bmmim][Br], where the C-2 proton is absent. Together, these confirmed the essential role of the acidic C2 hydrogen and formation of superoxide radical in the reaction mechanism. In order to investigate whether species **I** and **II** are in equilibrium (perhaps with BiBr_3_), we added BiBr_3_ (1 mmol) in a stirred mixture of Bi (1 mmol), allyl bromide (1.2 mmol) in [bmim][Br] (2 mL), stirred for additional 0.5 h, and ^1^H NMR spectrum of an aliquot taken from the reaction mixture was recorded in CD_2_Cl_2_ at −70 °C. However, no change in the stoichiometric ratio of species **I** and **II** was observed compared to what we observed in absence of added BiBr_3_. This invariably indicated that the species **I** and **II** are not in equilibrium, and are generated individually. The active species **I** and **II** reacted with the aldehydes to form the homoallylic alcohols, along with BiOBr, confirmed by the powder XRD analysis of the light yellow precipitate. Earlier, it has been reported [38,43] that the organobismuth halide generated in situ may act as a Lewis acid activator for the faster production of linear homoallylic alcohols. In the present case also, such a mechanism cannot be excluded.

## Conclusion

In conclusion, we have demonstrated a metal-activator free, practically viable and operationally simple protocol for the Bi-mediated Barbier-type allylation of aldehydes in [bmim][Br] for the first time. To the best of our knowledge, Bi-mediated allylation of aldehydes has never been attempted in an RTIL. The generality of the protocol was established by subjecting a variety of aldehydes to allylation. Moreover, we have probed the active allylbismuth species generated in situ using ^1^H VT-NMR, and have proposed a plausible mechanism for its formation.

## Supporting Information

File 1 Experimental details and analytical data for products **2a**–**2l'**.

File 2 NMR spectra for products **2a**–**2l'** and showing generation of species **I** and **II** in situ.
