# Supplementary material for: Bi-mediated allylation of aldehydes in [bmim][Br]: a mechanistic investigation
Source: Beilstein J Org Chem. 2018 Aug 22;14:2198–203. doi: 10.3762/bjoc.14.193 (PMC6122318; doi:10.3762/bjoc.14.193)
Supplement: File 1 — Experimental details and analytical data for products 2a–2l'. [file Beilstein_J_Org_Chem-14-2198-s001.pdf]

Supporting Information File 2

for

Bi-mediated allylation of aldehydes in [bmim][Br]: a mechanistic investigation

Mrunesh Koli[1], Sucheta Chatterjee[1], Subrata Chattopadhyay[1] and Dibakar Goswami[1,2*]

Address: [1]Bio-Organic Division, Bhabha Atomic Research Centre, Mumbai - 400 085, India and [2]Homi Bhabha National Institute, Training School Complex, Anushakti Nagar, Mumbai -400 094, India

Email: Dibakar Goswami - dibakarg@barc.gov.in

*Corresponding author

Experimental details and analytical data

All chemicals were procured from Sigma Aldrich and used as received. Other reagents were of AR grade. [bmim][Br] was synthesized following the procedure reported earlier [1]. The organic extracts were dried over anhydrous Na_2SO_4. The IR spectra were recorded as films with a BRUKER Tensor II spectrophotometer. The ^{1}H and ^{13}C NMR spectra were recorded with a Bruker 500 MHz or a Varian 500 MHz NMR spectrometer. For characterization, the NMR spectra were processed using Bruker TOPSPIN software or ACD/1D NMR Processor .

General procedure for Barbier-type allylation in aqueous or organic media. To a mixture of the aldehyde and allyl bromide in different solvents/solvent mixtures (10 mL each) was added finely powdered Bi metal (amounts specified in Table 1). After stirring for the duration mentioned in Table 1, the mixture was filtered, extracted with EtOAc (3 × 10 mL) and dried. The organic extract was evaporated in vacuo and the residue was purified by column chromatography (silica gel, 0–20% ethyl acetate-hexane) to give the respective products. In some case suitable metal activators (aqueous KF or ultrasonic irradiation) were also used. All reactions were carried at 3 mM scale.

Typical procedure for the Bi-mediated allylation reaction in RTILs. A mixture of finely powdered Bi metal and allyl bromide (quantities specified in Table 2) in the RTIL (2 mL/mmol) was stirred at room temperature for 0.5 h, followed by addition of the aldehyde. The reaction mixture was stirred at room temperature till completion of the reaction (cf. TLC, time

specified in Table 2). The mixture was extracted with Et_2O (3 × 10 mL), dried and the ether extract evaporated in vacuo. The residue was purified by column chromatography (silica gel, 0–20% EtOAc/hexane) to obtain the respective products.

Monitoring of in-situ generation of species I and II by NMR. A mixture of Bi (1 mmol) and allyl bromide (1.2 mmol) in [bmim][Br] (2 mL) was magnetically stirred at room temperature for 0.5 h. An aliquot (35 µL) of reaction mixture was taken, dissolved in CD_2Cl_2, and the ^{1}H NMR spectra recorded at different temperatures.

Analytical data for compounds 2a–2l'

Substrate	*Structure*	*Ref.*	*Characterization data*
**2a**	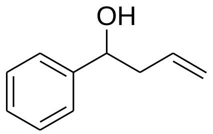	2	colorless liquid, IR (film): ν_{max} 3425 and 921 cm^{-1}; ^{1}H NMR[2] (500 MHz, $CDCl_3$): δ 2.51-2.54 (m, 2H), 2.56 (broad s, 1H), 4.71 (t, *J* = 6.5 Hz, 1H), 5.13-5.19 (m, 2H), 5.78-5.86 (m, 1H), 7.30-7.32 (m, 1H), 7.35-7.38 (m, 4H); ^{13}C NMR (125 MHz, $CDCl_3$): δ 43.6, 73.2, 118.0, 125.7, 127.3, 128.2, 134.4, 143.8.
**2b**	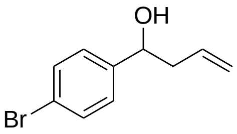	2	colorless liquid, IR (film): ν_{max} 3423 and 923 cm^{-1}; ^{1}H NMR[2] (500 MHz, $CDCl_3$): δ 2.38 (broad s, 1H), 2.40-2.50 (m, 2H), 4.66 (dd, *J* = 7.5 and 5.5 Hz, 1H), 5.12-5.16 (m, 2H), 5.71-5.80 (m, 1H), 7.20 (d, *J* = 8.0 Hz, 2H), 7.45 (d, *J* = 8.0 Hz, 2H); ^{13}C NMR (125 MHz, $CDCl_3$): δ 43.7, 72.5, 118.6, 121.1, 127.5, 131.3, 133.8,

			142.7.
**2c**	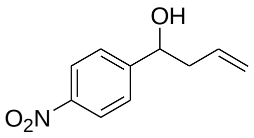	2	colorless liquid, IR (film): ν_{max} 3443 and 926 cm^{-1}; ^{1}H NMR[2] (500 MHz, $CDCl_3$): δ 2.41-2.47 (m, 2H), 2.51-2.56 (m, 1H), 4.84 (dd, *J* = 8.0 and 4.5 Hz, 1H), 5.13-5.17 (m, 2H), 5.72-5.80 (m, 1H), 7.50 (d, *J* = 8.5 Hz, 2H), 8.16 (d, *J* = 8.5 Hz, 2H); ^{13}C NMR (125 MHz, $CDCl_3$): δ 43.7, 72.1, 119.4, 123.5, 126.5, 133.2, 147.1, 151.1.
**2d**	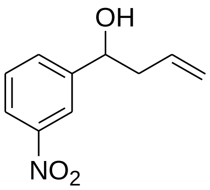	2	colorless liquid, IR (film): ν_{max} 3441 and 927 cm^{-1}; ^{1}H NMR[2] (500 MHz, $CDCl_3$): δ 2.43-2.49 (m, 1H), 2.51-2.56 (m, 1H), 2.68 (broad s, 1H), 4.83 (dd, *J* = 8.0 and 4.5 Hz, 1H), 5.12-5.15 (m, 2H), 5.72-5.80 (m, 1H), 7.48 (t, *J* = 8.0 Hz, 1H), 7.66 (d, *J* = 7.5 Hz, 1H), 8.07 (dd, *J* = 8.5 and 2.0 Hz, 1H), 8.19 (t, *J* = 2.0 Hz 1H); ^{13}C NMR (125 MHz, $CDCl_3$): δ 43.7, 72.0, 119.2, 120.7, 122.2, 129.2, 131.9, 133.2, 145.9, 148.1.
**2e**	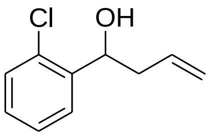	3	Light yellow oil, IR (film): ν_{max} 3420 and 918 cm^{-1}; ^{1}H NMR[3] (500 MHz, $CDCl_3$): δ 2.17 (broad s, 1H), 2.36-2.42 (m, 1H), 2.62-2.67 (m, 1H), 5.16-5.22 (m, 3H), 5.84-5.92 (m, 1H), 7.19-7.23 (m, 1H), 7.28-7.35 (m, 2H), 7.57 (dd, *J* = 7.5 and 1.5 Hz, 1H); ^{13}C NMR (125 MHz, $CDCl_3$): δ 42.0, 69.6, 118.6, 127.0, 127.1, 128.4, 129.4, 131.7, 134.2, 141.1.
**2f**	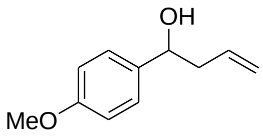	2	colorless liquid, IR (film): ν_{max} 3426 and 919 cm^{-1}; ^{1}H NMR[2] (500 MHz, $CDCl_3$): δ 2.37 (broad s, 1H), 2.47-2.50 (m, 2H), 3.78 (s, 3H), 4.65 (t, *J* = 6.5 Hz, 1H), 5.09-5.15 (m, 2H), 5.74-5.82 (m, 1H), 6.87 (d, *J* = 8.5 Hz, 2H), 7.26 (d, *J* = 8.5 Hz, 2H); ^{13}C NMR (125 MHz, $CDCl_3$): δ 43.5, 55.1, 72.9, 113.6, 117.8, 127.0, 134.5, 136.0, 158.8.

**2g**	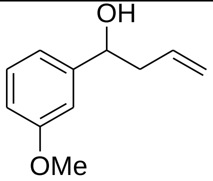	2	colorless liquid, IR (film): ν_{max} 3453 and 923 cm^{-1}; ^{1}H NMR[2] (500 MHz, $CDCl_3$): δ 2.28 (broad s, 1H), 2.48-2.52 (m, 2H), 3.81 (s, 3H), 4.65-4.70 (m, 1H), 5.11-5.18 (m, 2H), 5.76-5.85 (m, 1H), 6.82 (dd, J = 8.0 and 2.5 Hz, 1H), 6.87-6.93 (m, 2H), 7.24-7.28 (m, 1H); ^{13}C NMR (125 MHz, $CDCl_3$): δ 43.6, 55.1, 73.1, 111.2, 112.9, 113.7, 118.1, 127.0, 129.3, 134.4, 145.6, 159.6.
**2h**	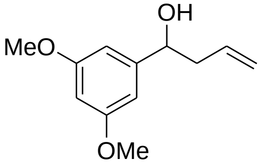	4	colorless liquid, IR (film): ν_{max} 3425 and 927 cm^{-1}; ^{1}H NMR[4] (500 MHz, $CDCl_3$): δ 2.28 (broad s, 1H), 2.46-2.51 (m, 2H), 3.78 (s, 6H), 4.65 (dd, J = 8.0 and 5.0 Hz, 1H), 5.12-5.18 (m, 2H), 5.76-5.85 (m, 1H), 6.37 (t, J = 2.0 Hz, 1H), 6.51 (d, J = 2.0 Hz, 2H); ^{13}C NMR (125 MHz, $CDCl_3$): δ 43.6, 55.2, 73.3, 99.3, 103.7, 118.2, 134.4, 146.5, 160.7.
**2i**	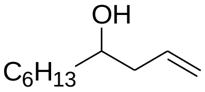	5	colorless liquid, IR (film): ν_{max} 3425 and 926 cm^{-1}; ^{1}H NMR[5] (500 MHz, $CDCl_3$): δ 0.88 (t, J = 6.0 Hz, 3H), 1.25-1.33 (m, 7H), 1.42-1.48 (m, 3H), 1.73 (broad s, 1H), 2.11-2.17 (m, 1H), 2.27-2.33 (m, 1H), 3.62-3.67 (m, 1H), 5.11-5.15 (m, 2H), 5.79-5.87 (m, 1H); ^{13}C NMR (125 MHz, $CDCl_3$): δ 14.0, 22.6, 25.6, 29.3, 31.8, 36.8, 41.9, 70.7, 118.0, 134.9.
**2j**	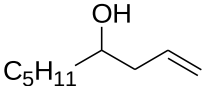	2	colorless liquid, IR (film): ν_{max} 3426 and 921 cm^{-1}; ^{1}H NMR[2] (500 MHz, $CDCl_3$): δ 0.90 (t, J = 7.0 Hz, 3H), 1.29-1.35 (m, 6H), 1.42-1.48 (m, 2H), 1.68 (broad s, 1H), 2.12-2.18 (m, 1H), 2.28-2.33 (m, 1H), 3.64-3.67 (m, 1H), 5.12-5.16 (m, 2H), 5.80-5.88 (m, 1H); ^{13}C NMR (125 MHz, $CDCl_3$): δ 14.0, 22.6, 25.3, 31.8, 36.8, 41.9, 70.7, 118.0, 134.9.
**2k**	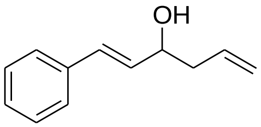	5	colorless liquid, IR (film): ν_{max} 3425 and 923 cm^{-1}; ^{1}H NMR[5] (500 MHz, $CDCl_3$): δ 1.86 (broad s, 1 H), 2.37-2.54 (two m, 2H), 4.35-4.39 and 4.74-4.77 (two m, 1H), 5.15-5.22 (m, 2H), 5.83-5.92 (m, 1H), 6.26 (dd, J = 16.0 and 6.5 Hz, 1H), 6.62 (d, J = 16.0 Hz, 1H), 7.25-7.41 (m, 5H); ^{13}C NMR (125 MHz, $CDCl_3$): δ 42.0, 43.8, 71.7, 73.3, 118.5, 125.8, 126.5, 127.5, 127.6, 128.4, 128.6, 130.4, 131.6, 134.0, 136.7.

**2l**	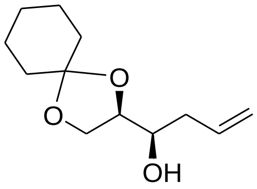	6	colorless liquid, $[\alpha]_D^{24}$ +5.7 (*c* 1.10, $CHCl_3$) (lit.[6] $[\alpha]_D^{23}$ +5.3 (*c* 1.34, $CHC1_3$)); IR (film): ν_{max} 3424 and 927 cm^{-1}; ^1H NMR (500 MHz, $CDCl_3$): δ 1.29-1.32 (m, 2H), 1.46-1.55 (m, 8H), 2.16 (t, *J* = 6.5 Hz, 2H), 2.22 (broad s, 1H), 3.47-3.50 (m, 1H), 3.63-3.66 (m, 1H), 3.89-3.95 (m, 2H), 5.01-5.06 (m, 2H), 5.73-5.81 (m, 1H); ^{13}C NMR (125 MHz, $CDCl_3$): δ 23.7, 23.9, 25.0, 34.7, 36.2, 38.2, 65.6, 71.5, 78.0, 109.8, 117.6, 134.0.
**2l'**	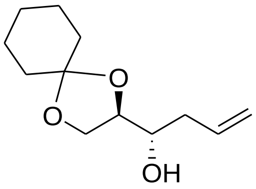	6	colorless liquid, $[\alpha]_D^{24}$ +10.1 (*c* 1.30, $CHCl_3$) (lit.[6] $[\alpha]_D^{25}$ +10.2 (*c* 1.41, $CHCl_3$)); IR (film): ν_{max} 3426 and 926 cm^{-1}; ^1H NMR (500 MHz, $CDCl_3$): δ 1.36-1.39 (m, 2H), 1.57-1.61 (m, 8H), 2.09-2.22 (m merged with broad s, 2H), 2.29-2.34 (m, 1H), 3.74-3.78 (m, 1H), 3.90-3.92 (m, 1H), 3.97-3.99 (m, 2H), 5.11-5.16 (m, 2H), 5.79-5.87 (m, 1H); ^{13}C NMR (125 MHz, $CDCl_3$): δ 23.8, 24.0, 25.1, 34.8, 36.2, 37.6, 64.8, 70.5, 77.7, 109.6, 118.1, 134.1.

References

1. Goswami, D.; Koli, M. R.; Chatterjee, S.; Chattopadhyaya, S.; Sharma, A. *Org. Biomol. Chem.* **2017**, *15*, 3756-3774.
2. Freitas, J. J. R.; Couto, T. R.; Cavalcanti, I. H.; Freitas, J. C. R.; Oliveira, R. A.; Menezes, P. H. *Ultrason. Sonochem.* **2014**, *21*, 1609−1614.
3. Lidia, C. A.; Carles, R. E.; Laura, O. P.; Miquel, A. P. *ACS Catalysis* **2016**, *6*, 7647-7651.
4. White, J. D.; Shaw, S. *Org. Lett.* **2011**, *13*, 2488−2491.
5. Goswami, D.; Chattopadhyay, A.; Sharma, A.; Chattopadhyay, S. *J. Org. Chem.* **2012**, *77*, 11064–11070.
6. Chattopadhyay, A.; Mamdapur, V. R. *J. Org. Chem.* **1995**, *60*, 585−587.
