# Supplementary material for: Bi-mediated allylation of aldehydes in [bmim][Br]: a mechanistic investigation
Source: Beilstein J Org Chem. 2018 Aug 22;14:2198–203. doi: 10.3762/bjoc.14.193 (PMC6122318; doi:10.3762/bjoc.14.193)
Supplement: File 2 — NMR spectra for products 2a–2l' and showing generation of species I and II in situ. [file Beilstein_J_Org_Chem-14-2198-s002.pdf]

Supporting Information File 2

for

Bi-mediated allylation of aldehydes in [bmim][Br]: a mechanistic investigation

Mrunesh Koli[1], Sucheta Chatterjee[1], Subrata Chattopadhyay[1] and Dibakar Goswami[1,2*]

Address: [1]Bio-Organic Division, Bhabha Atomic Research Centre, Mumbai - 400 085, India and [2]Homi Bhabha National Institute, Training School Complex, Anushakti Nagar, Mumbai -400 094, India

Email: Dibakar Goswami - dibakarg@barc.gov.in

*Corresponding author

NMR SPECTRA

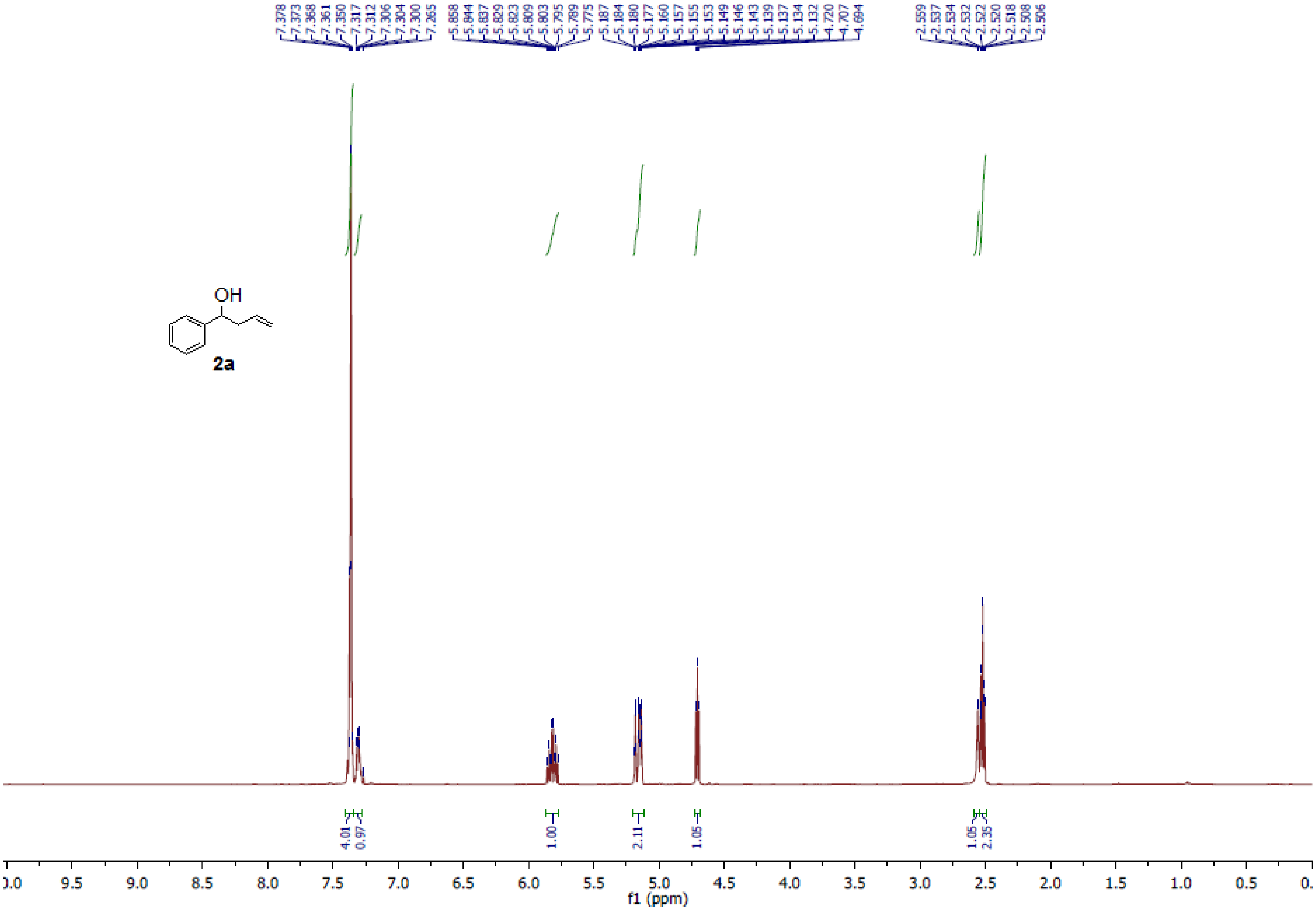
7.378
7.373
7.368
7.361
7.350
7.317
7.312
7.306
7.304
7.300
7.265
5.858
5.844
5.837
5.829
5.823
5.809
5.803
5.795
5.789
5.775
5.187
5.184
5.180
5.177
5.160
5.157
5.155
5.153
5.149
5.146
5.143
5.139
5.137
5.134
5.132
4.720
4.707
4.694
2.559
2.537
2.534
2.532
2.522
2.520
2.518
2.508
2.506
OH
2a
4.01
0.97
1.00
2.11
1.05
1.05
2.35
f1 (ppm)

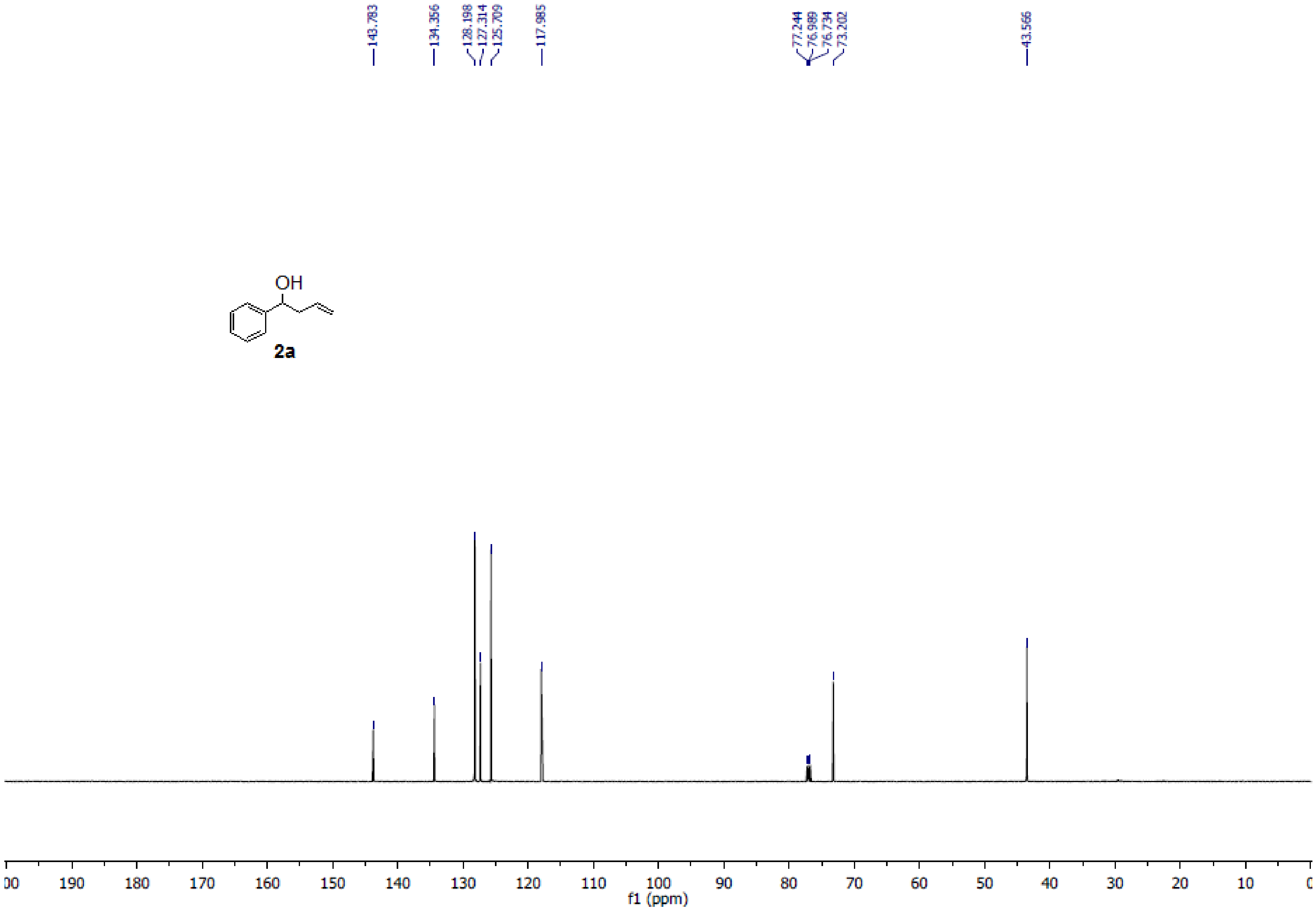
143.783
134.356
128.198
127.314
125.709
117.985
77.244
76.989
76.734
73.202
43.566
f1 (ppm)
OH

2a

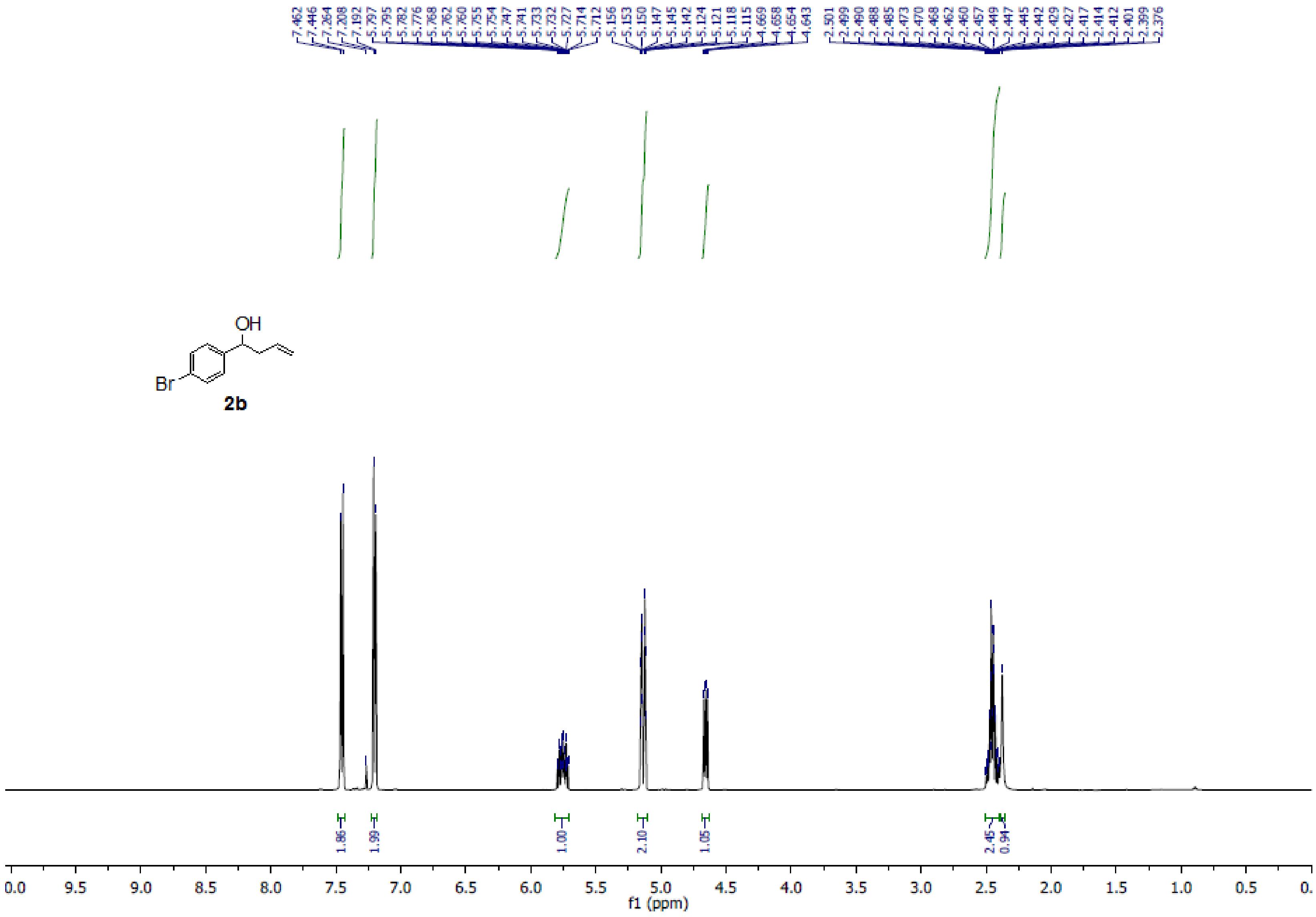

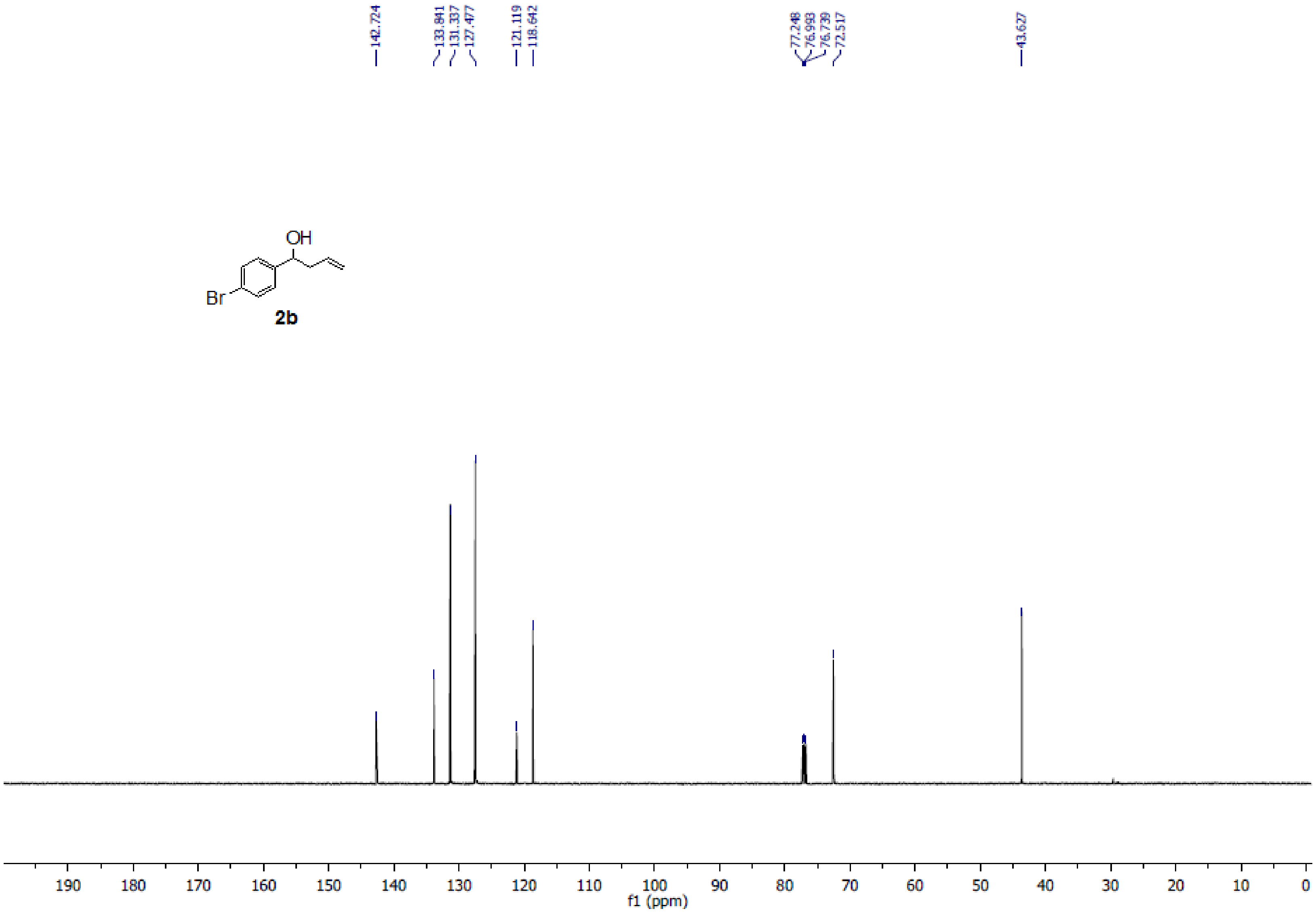

OH
Br
2b
142.724
133.841
131.337
127.477
121.119
118.642
77.248
76.993
76.739
72.517
43.627
190
180
170
160
150
140
130
120
110
100
90
80
70
60
50
40
30
20
10
0
f1 (ppm)

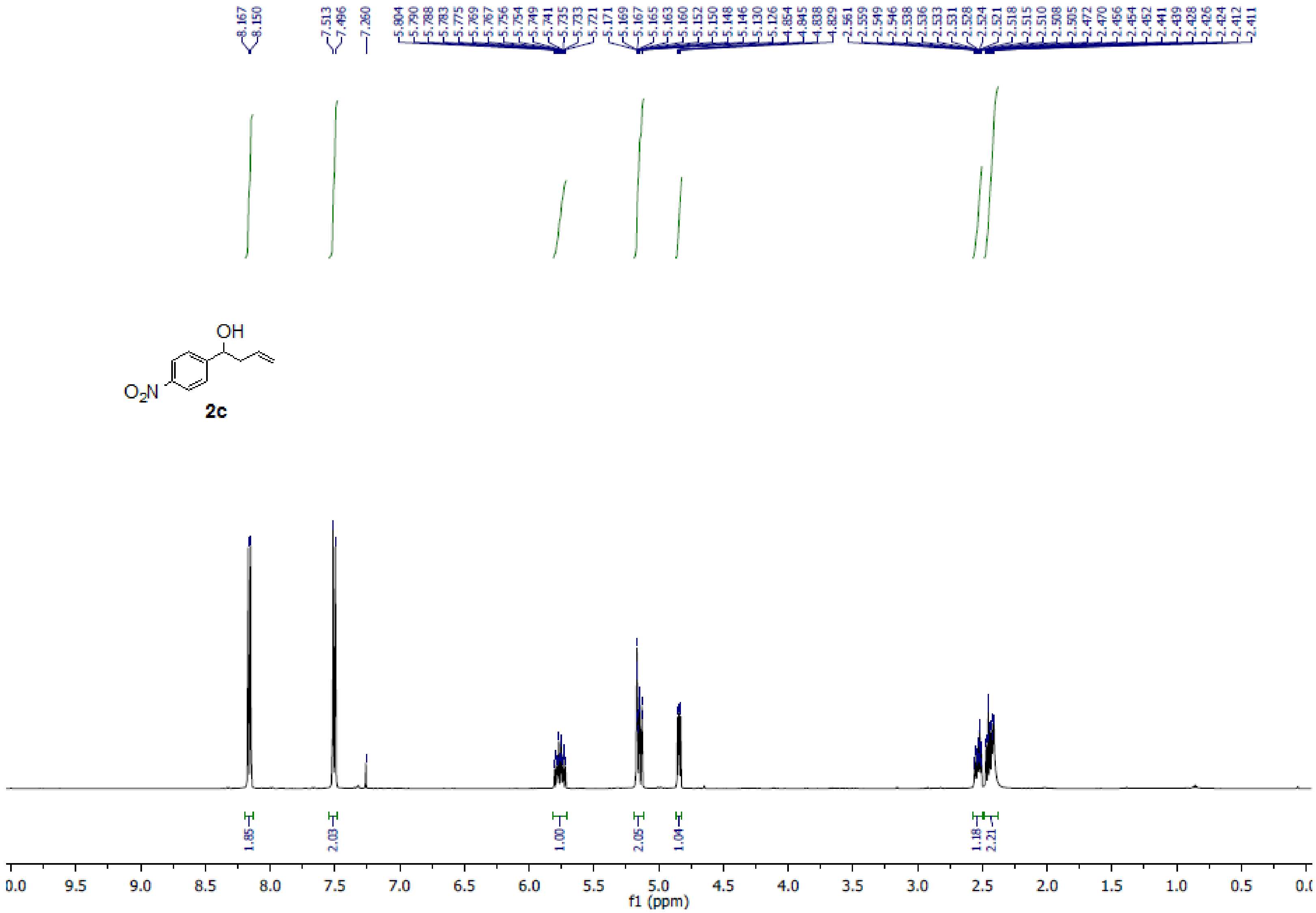
OH
O_2N
2c
f1 (ppm)

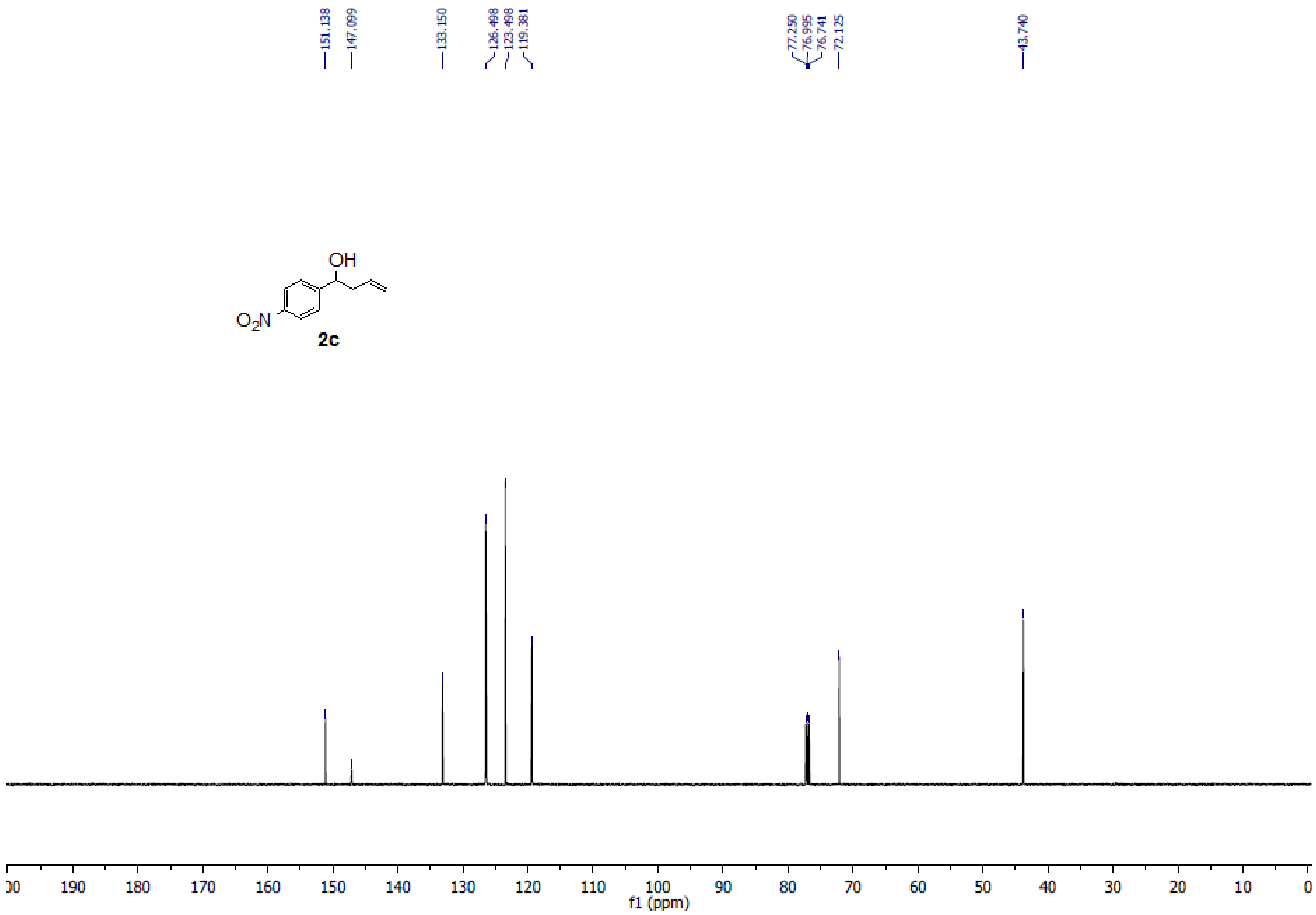
151.138
147.099
133.150
126.498
123.498
119.381
77.250
76.995
76.741
72.125
43.740
OH
O2N
2c
f1 (ppm)

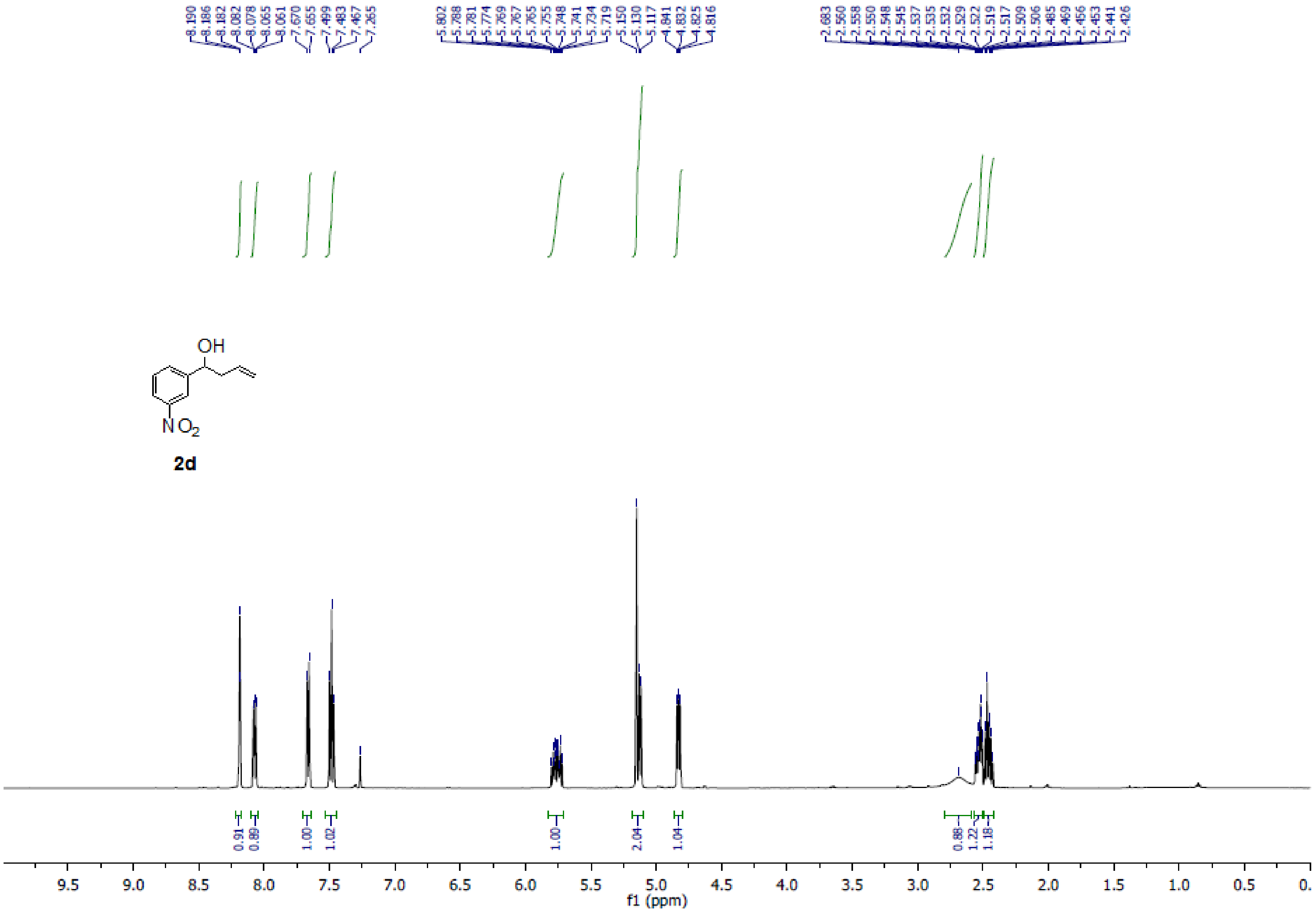

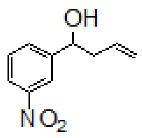
OH
NO2

2d

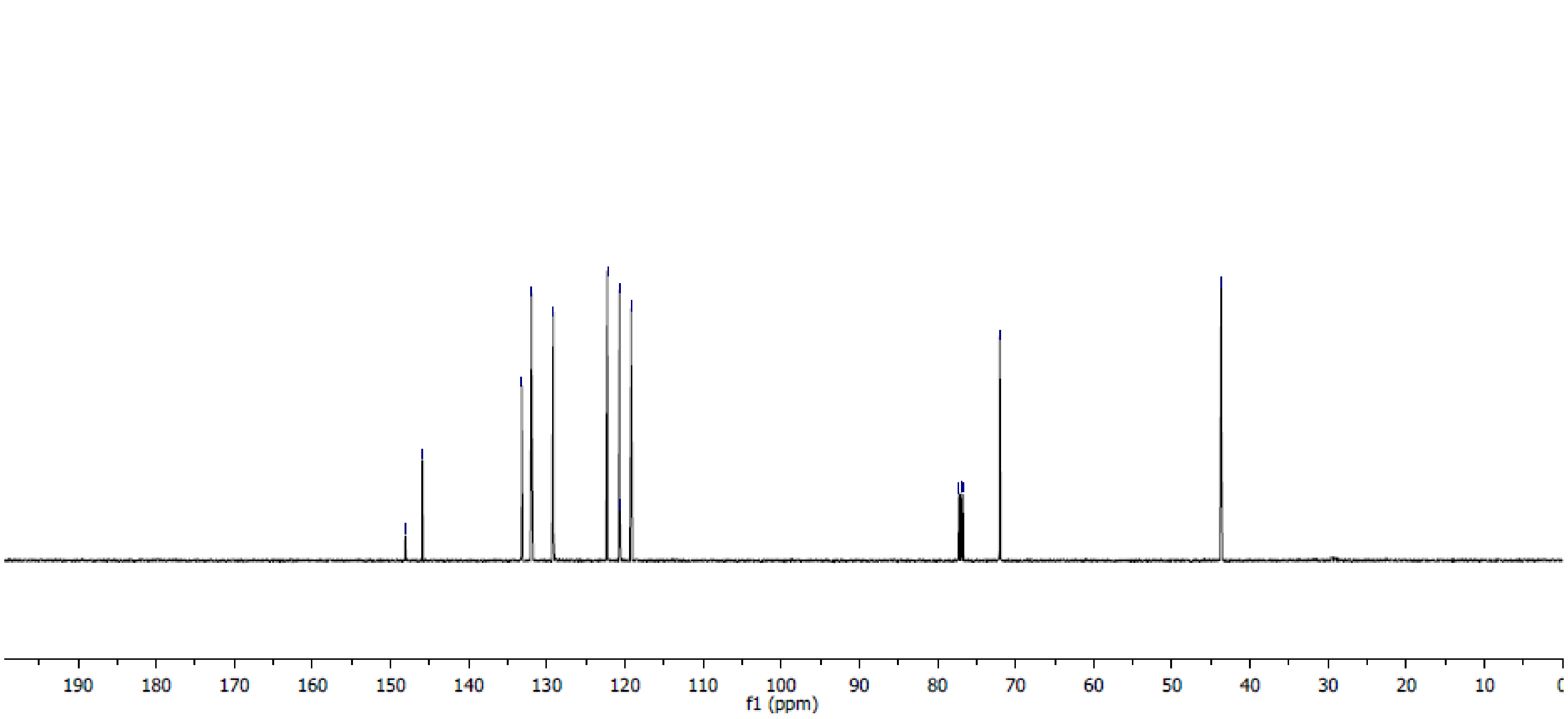
148.100
145.914
133.196
131.927
129.169
122.242
120.690
120.681
119.207
77.246
76.992
76.737
72.021
43.669
190
180
170
160
150
140
130
120
110
100
90
80
70
60
50
40
30
20
10
f1 (ppm)

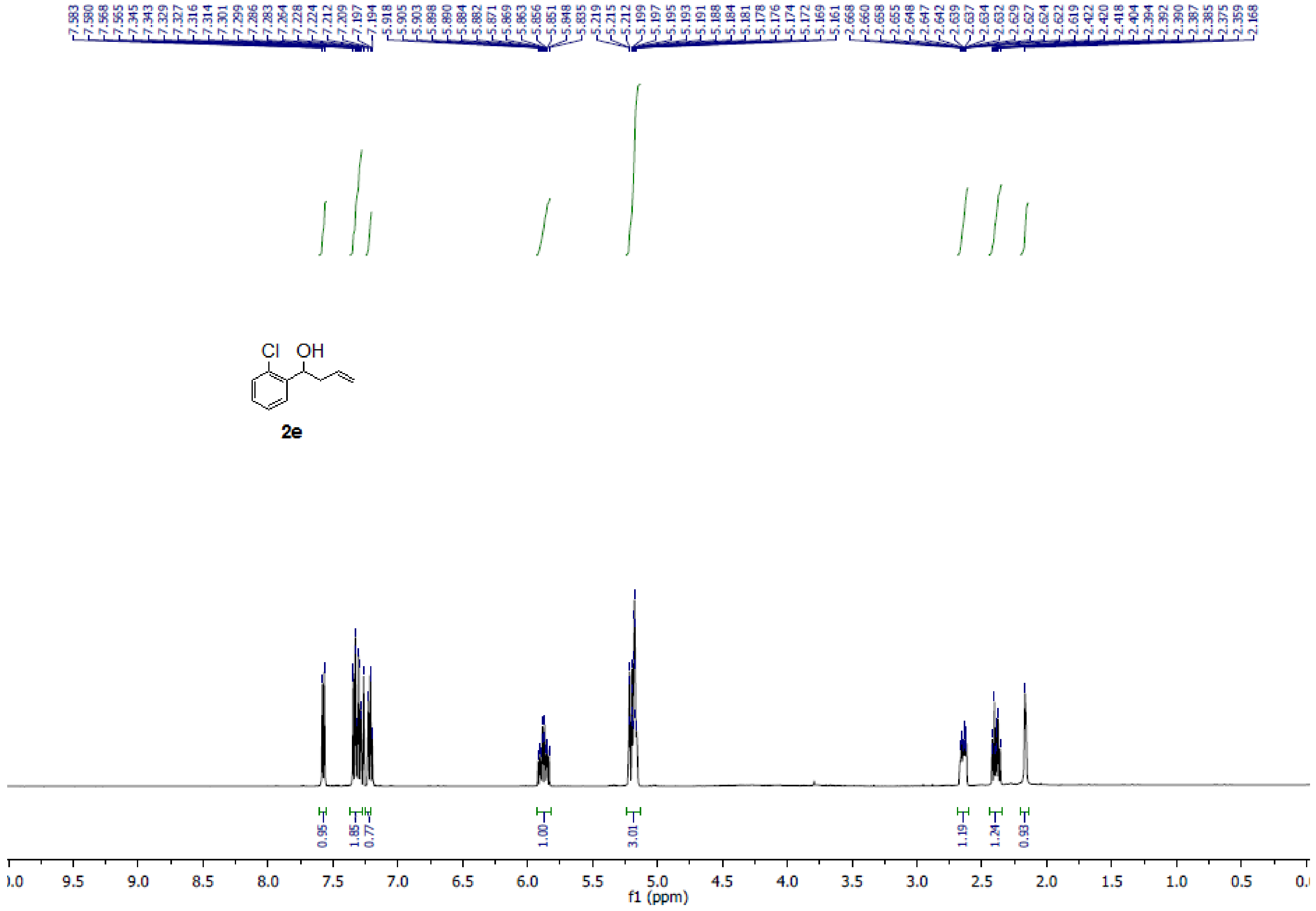
7.583 7.580 7.568 7.565 7.345 7.343 7.329 7.327 7.316 7.314 7.301 7.299 7.286 7.283 7.264 7.228 7.224 7.212 7.209 7.197 7.194
5.918 5.905 5.903 5.898 5.890 5.884 5.882 5.871 5.869 5.863 5.856 5.851 5.848 5.835
5.219 5.215 5.212 5.199 5.197 5.195 5.193 5.191 5.188 5.184 5.181 5.178 5.176 5.174 5.172 5.169 5.161
2.668 2.660 2.658 2.655 2.648 2.647 2.642 2.639 2.637 2.634 2.632 2.629 2.627 2.624 2.622 2.619 2.422 2.420 2.418 2.404 2.394 2.392 2.390 2.387 2.385 2.375 2.359 2.168
Cl
OH
2e
0.95
1.85
0.77
1.00
3.01
1.19
1.24
0.93
9.5
9.0
8.5
8.0
7.5
7.0
6.5
6.0
5.5
5.0
4.5
4.0
3.5
3.0
2.5
2.0
1.5
1.0
0.5
f1 (ppm)

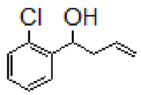
Cl
OH

2e

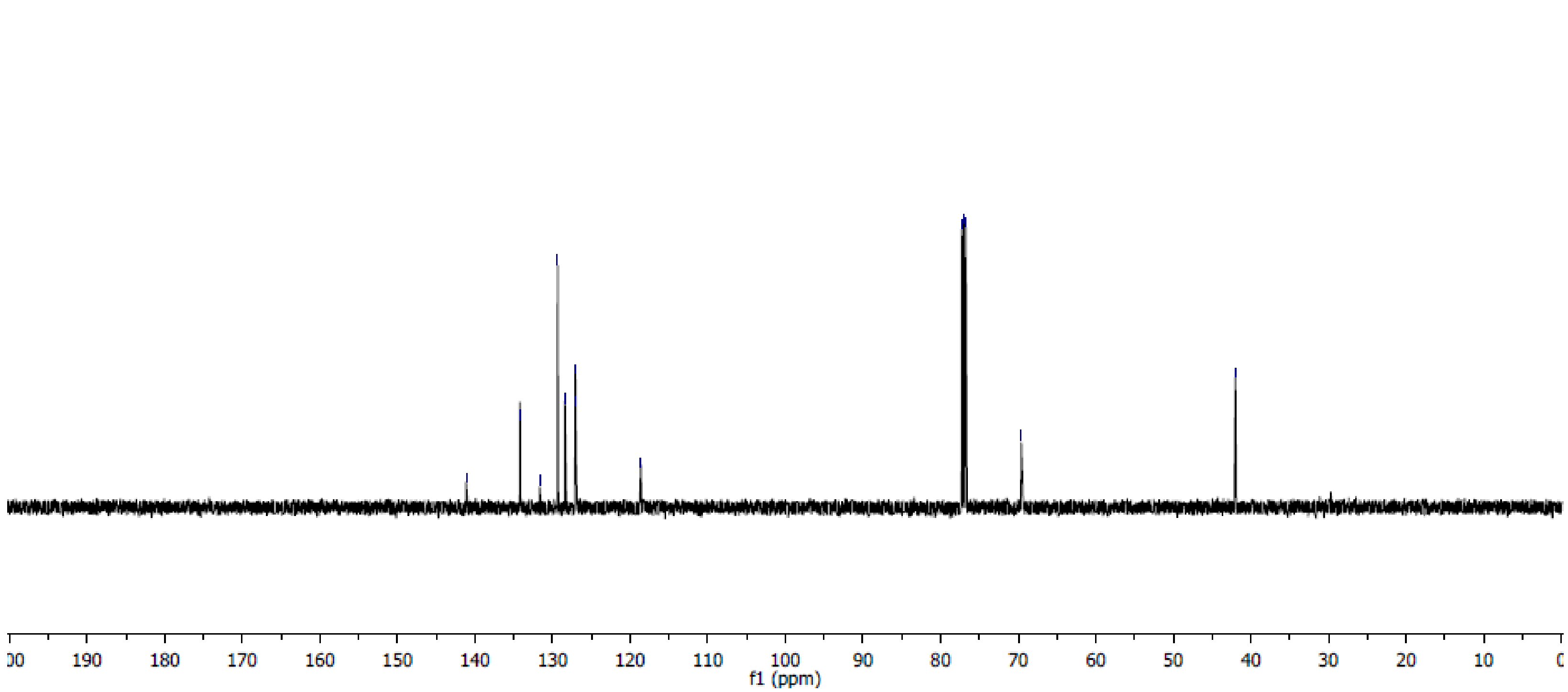
141.149
134.208
131.688
129.358
128.405
127.055
126.984
118.618
77.239
76.985
76.731
69.612
41.995
f1 (ppm)

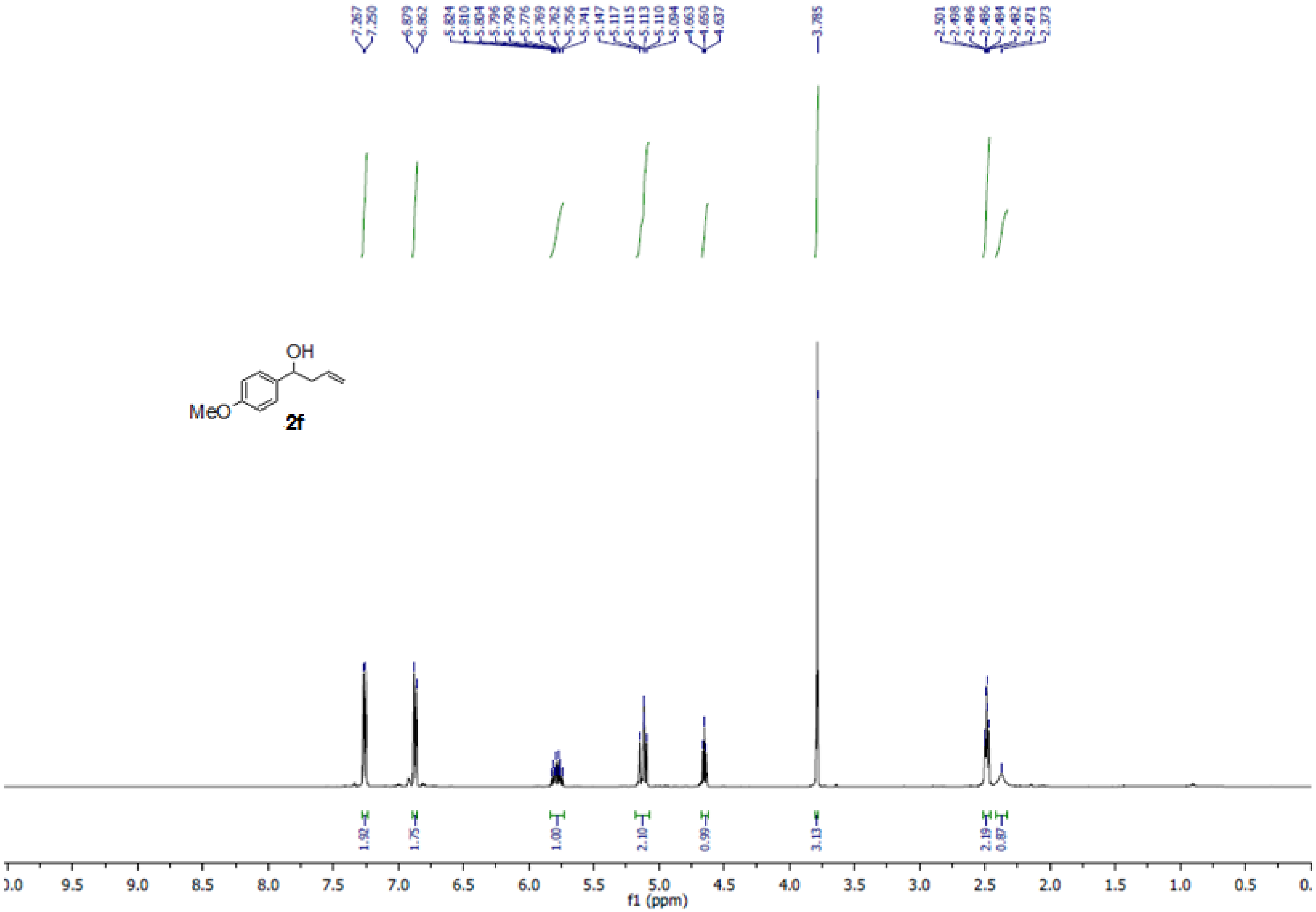
7.267
7.250
6.879
6.862
5.824
5.810
5.804
5.796
5.790
5.776
5.769
5.762
5.756
5.741
5.147
5.117
5.115
5.113
5.110
5.094
4.663
4.650
4.637
3.785
2.501
2.498
2.496
2.486
2.484
2.482
2.471
2.373
OH
MeO
2f
1.92
1.75
1.00
2.10
0.99
3.13
2.19
0.87
f1 (ppm)

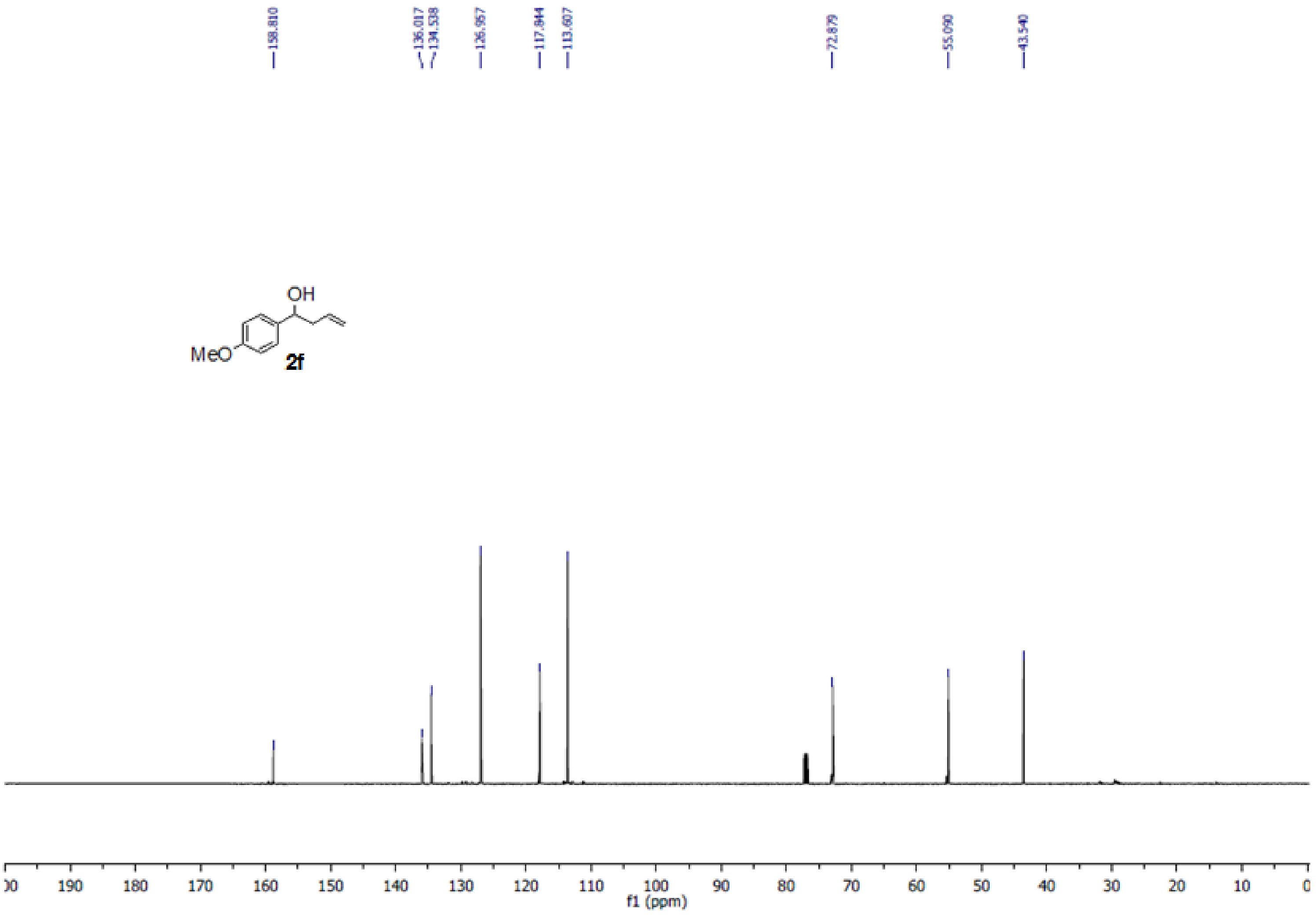
158.810
136.017
134.538
126.957
117.844
113.607
72.879
55.090
43.540
OH
MeO
2f
f1 (ppm)

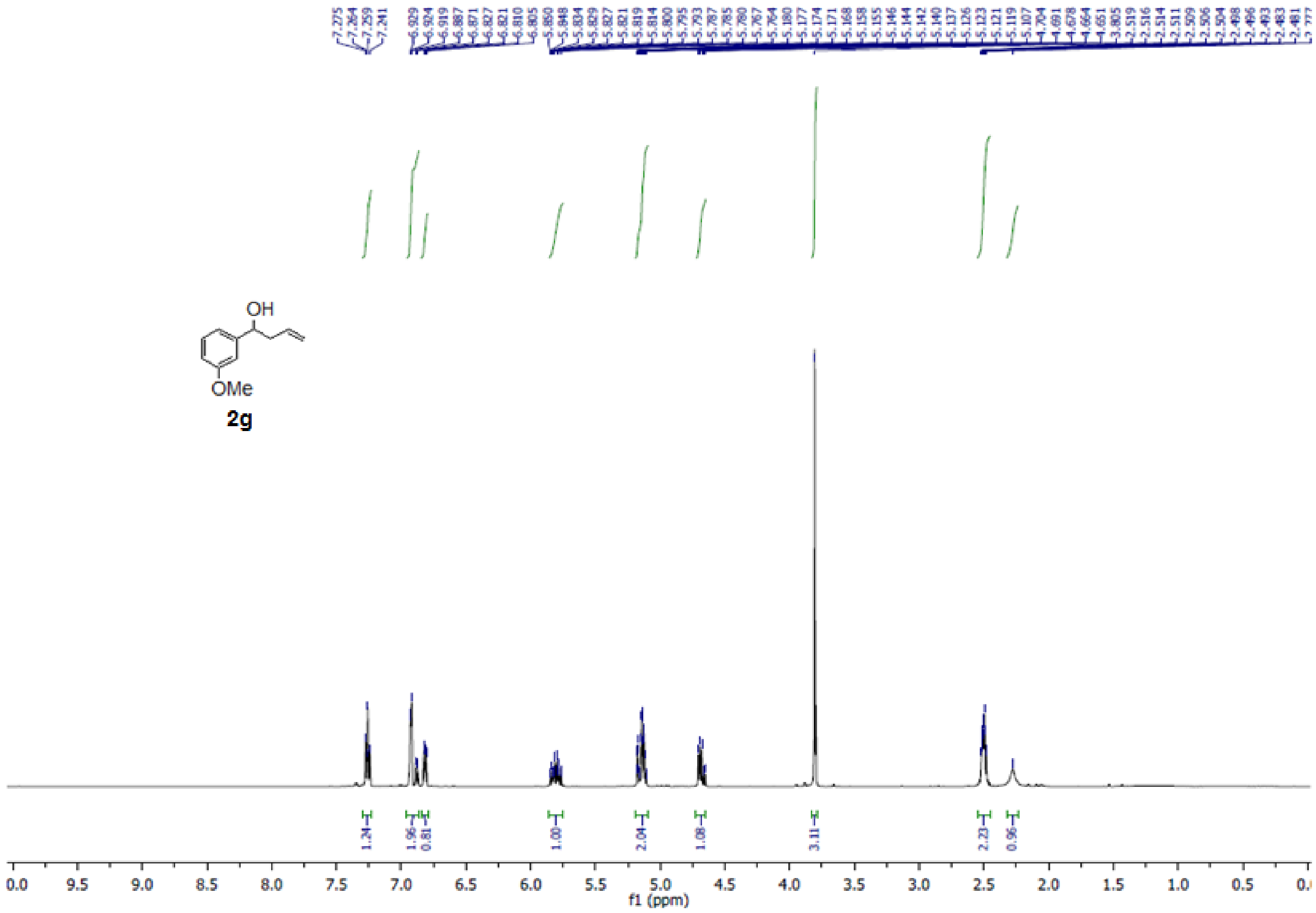

OH
OMe
2g
1.24
1.96
0.81
1.00
2.04
1.08
3.11
2.23
0.96
f1 (ppm)

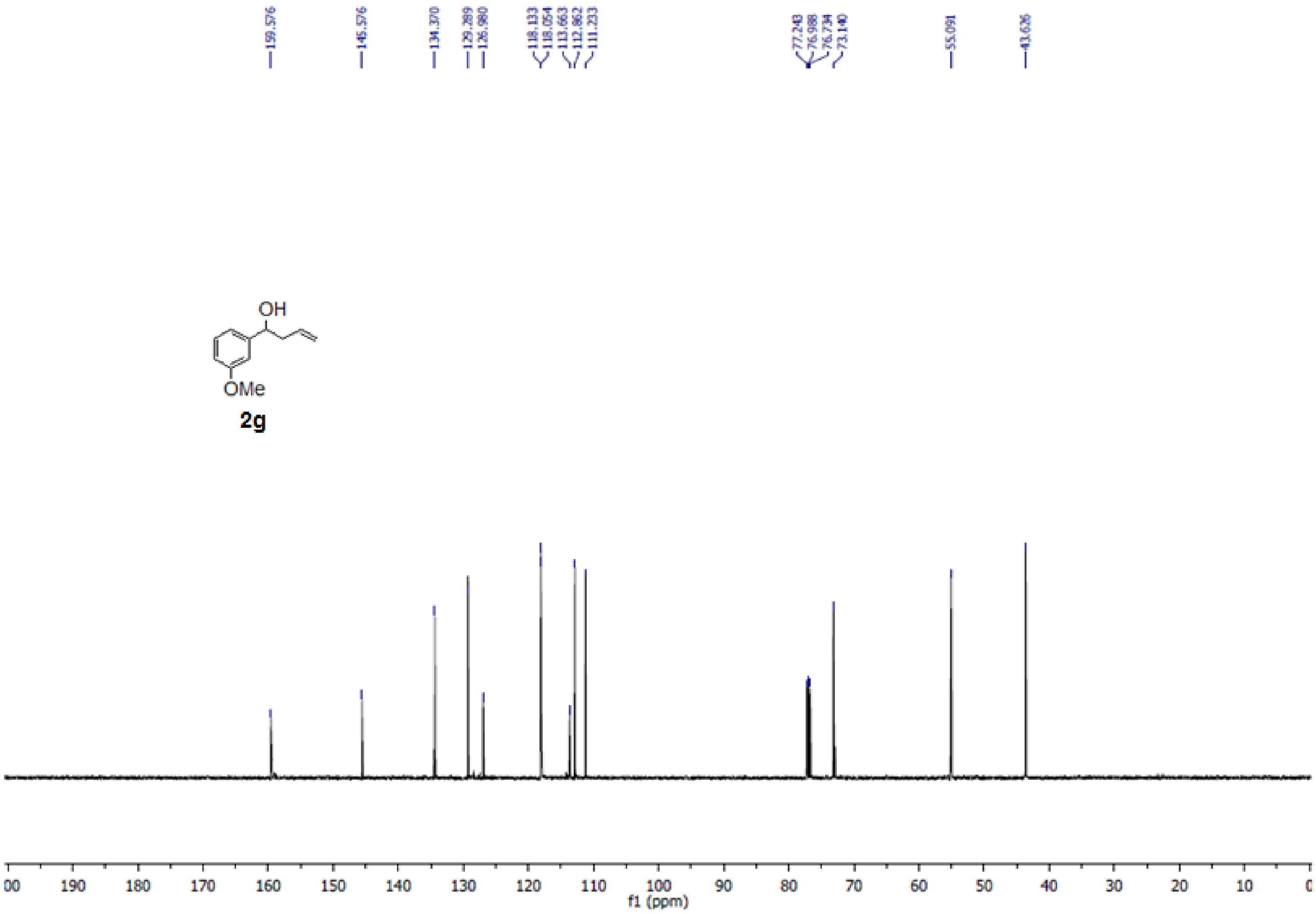
159.576
145.576
134.370
129.289
126.980
118.133
118.054
113.663
112.862
111.233
77.243
76.988
76.734
73.140
55.091
43.626
OH
OMe
f1 (ppm)

2g

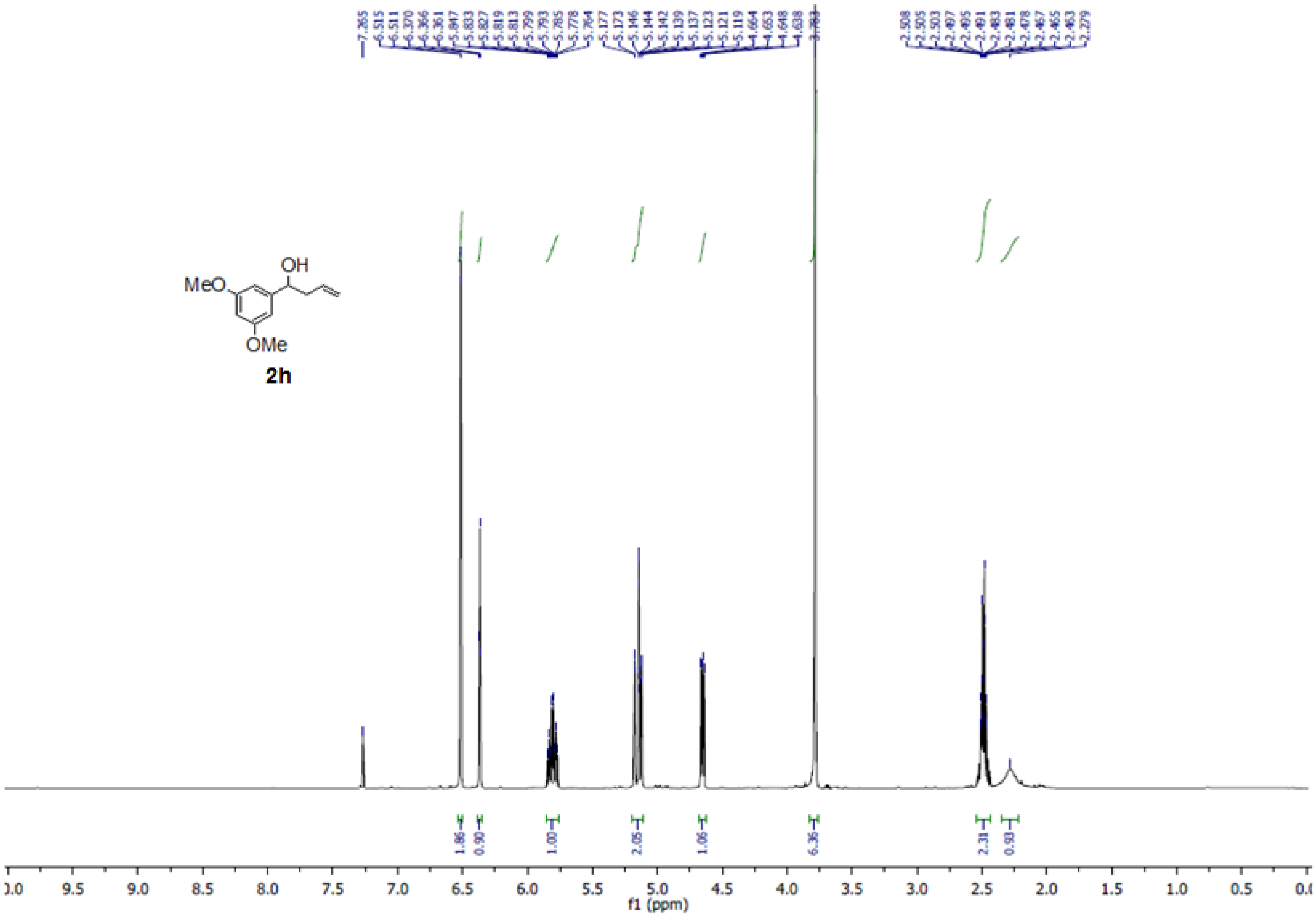

OH
MeO
OMe
2h
7.265
6.515
6.511
6.370
6.366
6.361
5.847
5.833
5.827
5.819
5.813
5.799
5.793
5.785
5.778
5.764
5.177
5.173
5.146
5.144
5.142
5.139
5.137
5.123
5.121
5.119
4.664
4.653
4.648
4.638
3.783
2.508
2.505
2.503
2.497
2.495
2.491
2.483
2.481
2.478
2.467
2.465
2.463
2.279
1.86
0.90
1.00
2.05
1.06
6.36
2.31
0.93
9.5
9.0
8.5
8.0
7.5
7.0
6.5
6.0
5.5
5.0
4.5
4.0
3.5
3.0
2.5
2.0
1.5
1.0
0.5
f1 (ppm)

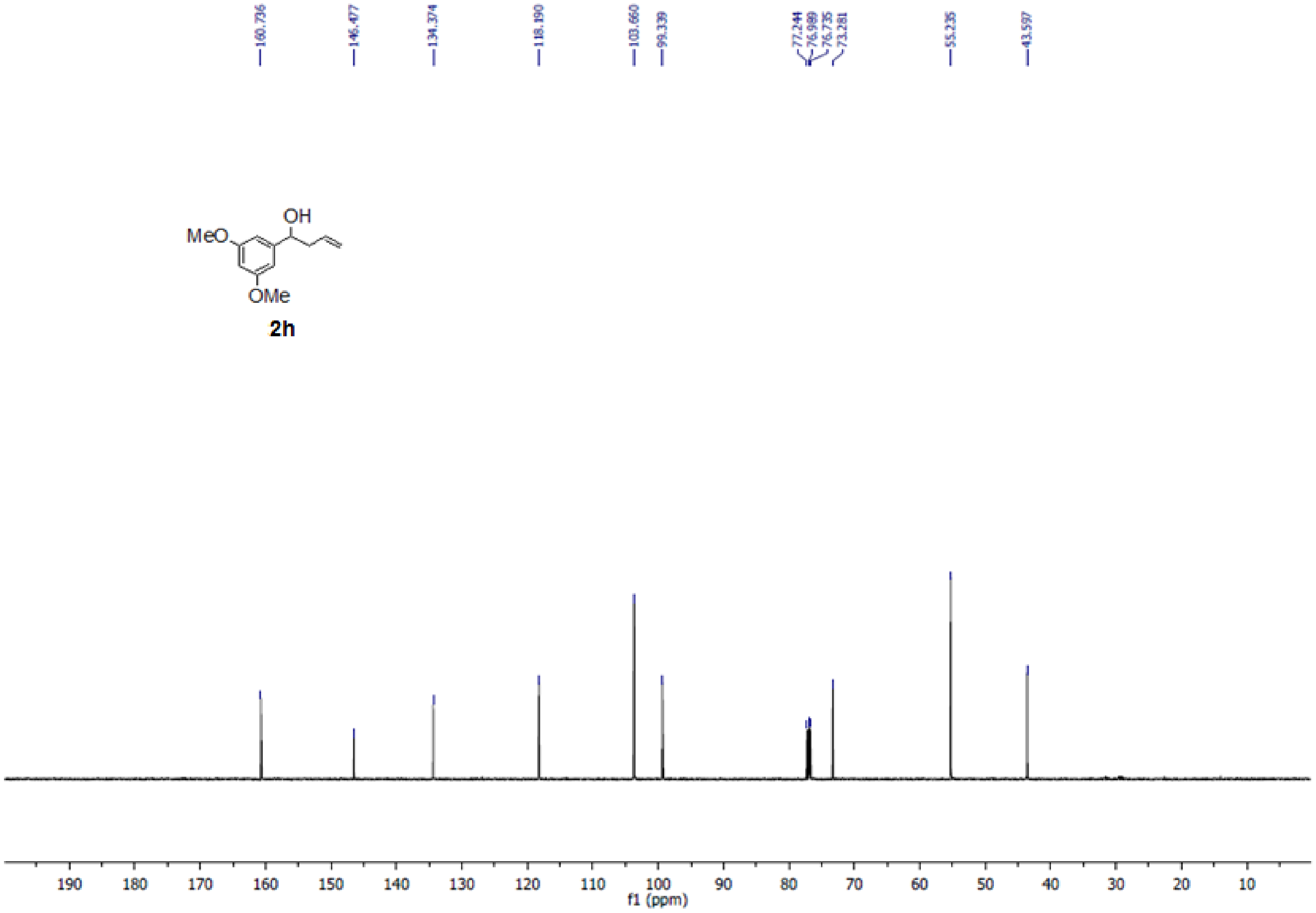

160.736
146.477
134.374
118.190
103.660
99.339
77.244
76.989
76.735
73.281
55.235
43.597
190 180 170 160 150 140 130 120 110 100 90 80 70 60 50 40 30 20 10
f1 (ppm)
OH
MeO
OMe

2h

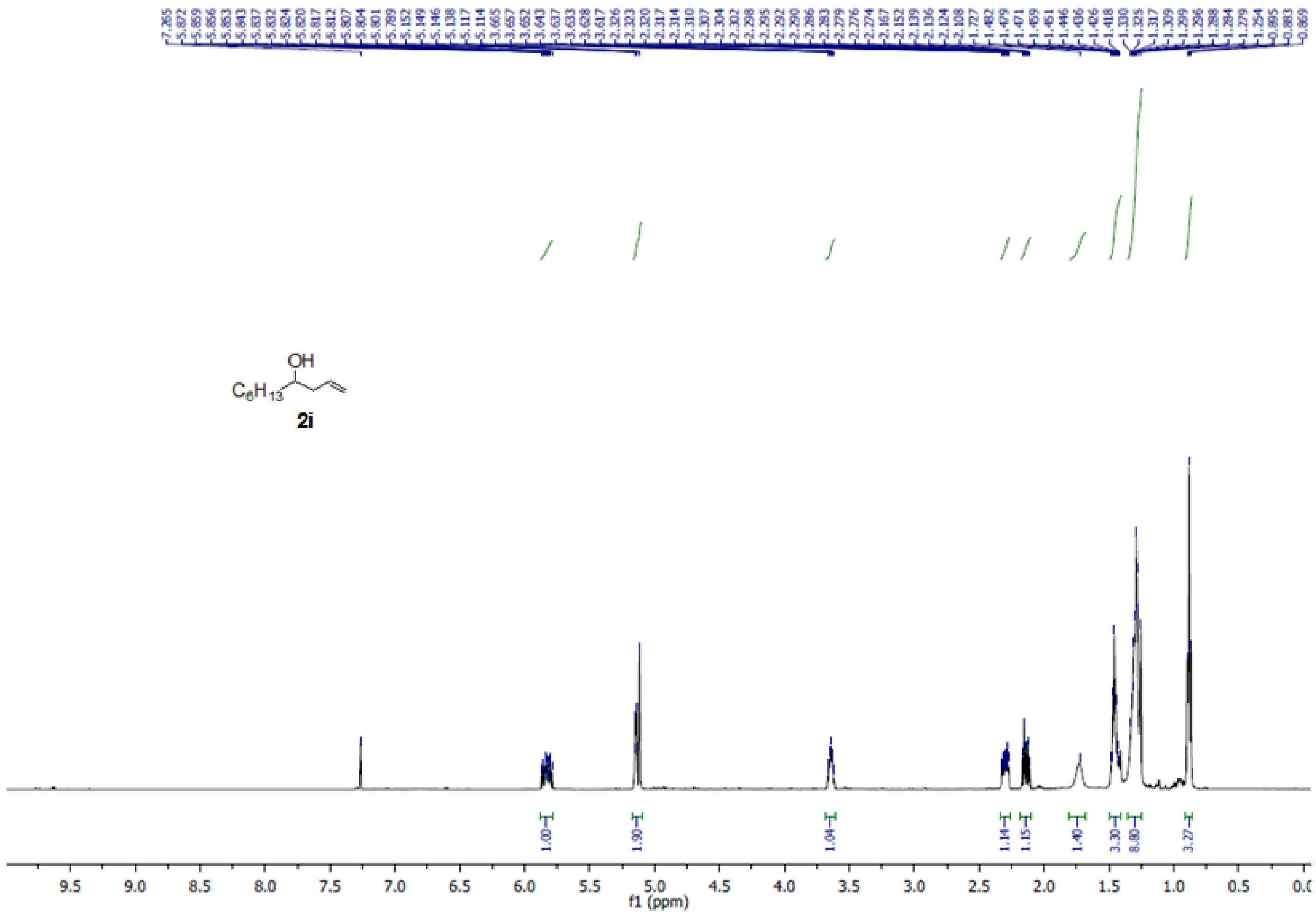
7.265
5.872
5.859
5.856
5.853
5.843
5.837
5.832
5.824
5.820
5.817
5.812
5.807
5.804
5.801
5.789
5.152
5.149
5.146
5.138
5.117
5.114
3.665
3.657
3.652
3.643
3.637
3.633
3.628
3.617
2.326
2.323
2.320
2.317
2.314
2.310
2.307
2.304
2.302
2.298
2.295
2.292
2.290
2.286
2.283
2.279
2.276
2.274
2.167
2.152
2.139
2.136
2.124
2.108
1.727
1.482
1.479
1.471
1.459
1.451
1.446
1.436
1.426
1.418
1.330
1.325
1.317
1.309
1.299
1.296
1.288
1.284
1.279
1.254
0.895
0.883
0.869
OH
C_6H_{13}
2i
1.00
1.90
1.04
1.14
1.15
1.40
3.30
8.80
3.27
9.5
9.0
8.5
8.0
7.5
7.0
6.5
6.0
5.5
5.0
4.5
4.0
3.5
3.0
2.5
2.0
1.5
1.0
0.5
0.0
f1 (ppm)

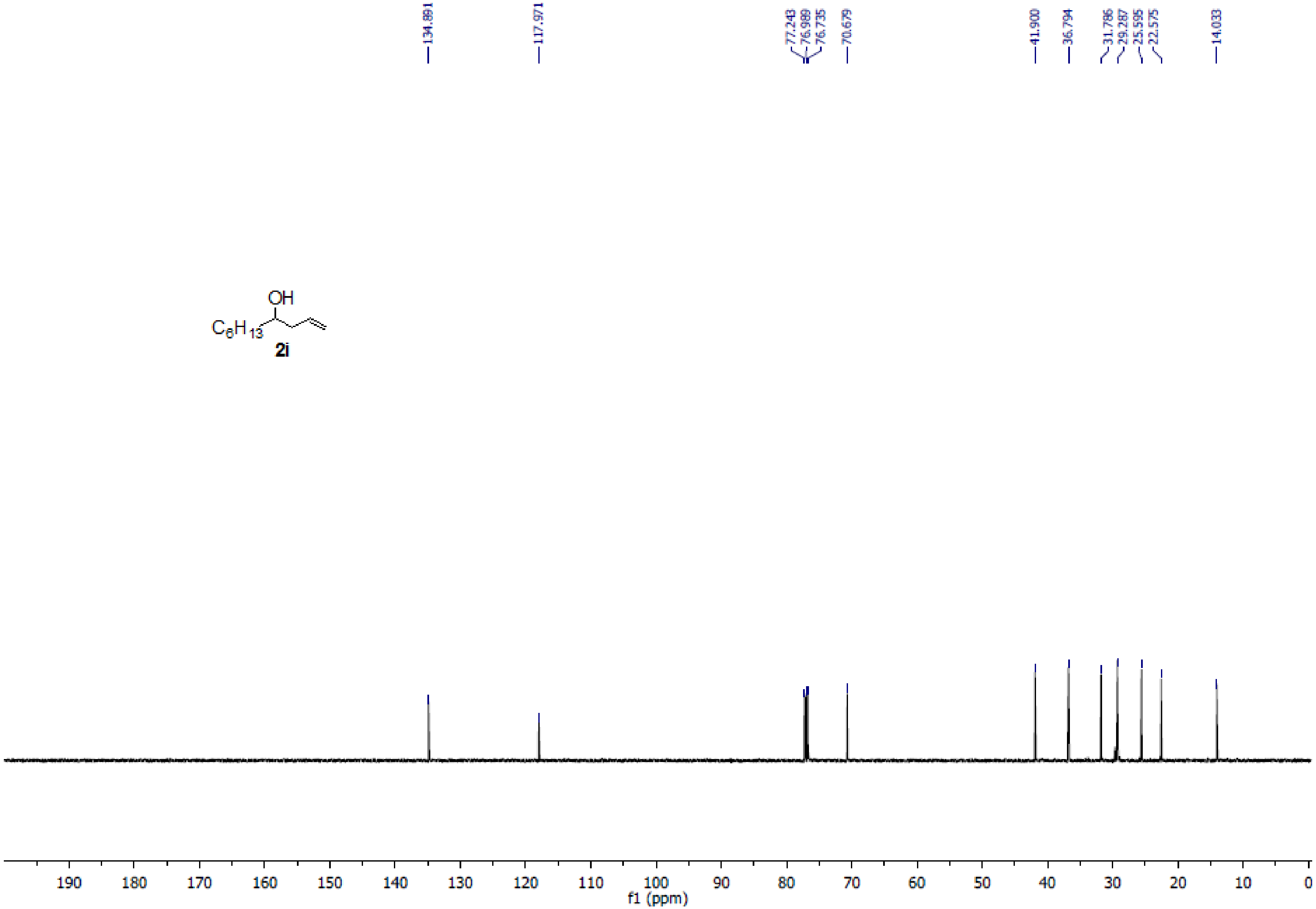

134.891
117.971
77.243
76.989
76.735
70.679
41.900
36.794
31.786
29.287
25.595
22.575
14.033
OH
C_6H_{13}
190 180 170 160 150 140 130 120 110 100 90 80 70 60 50 40 30 20 10 0
f1 (ppm)

2i

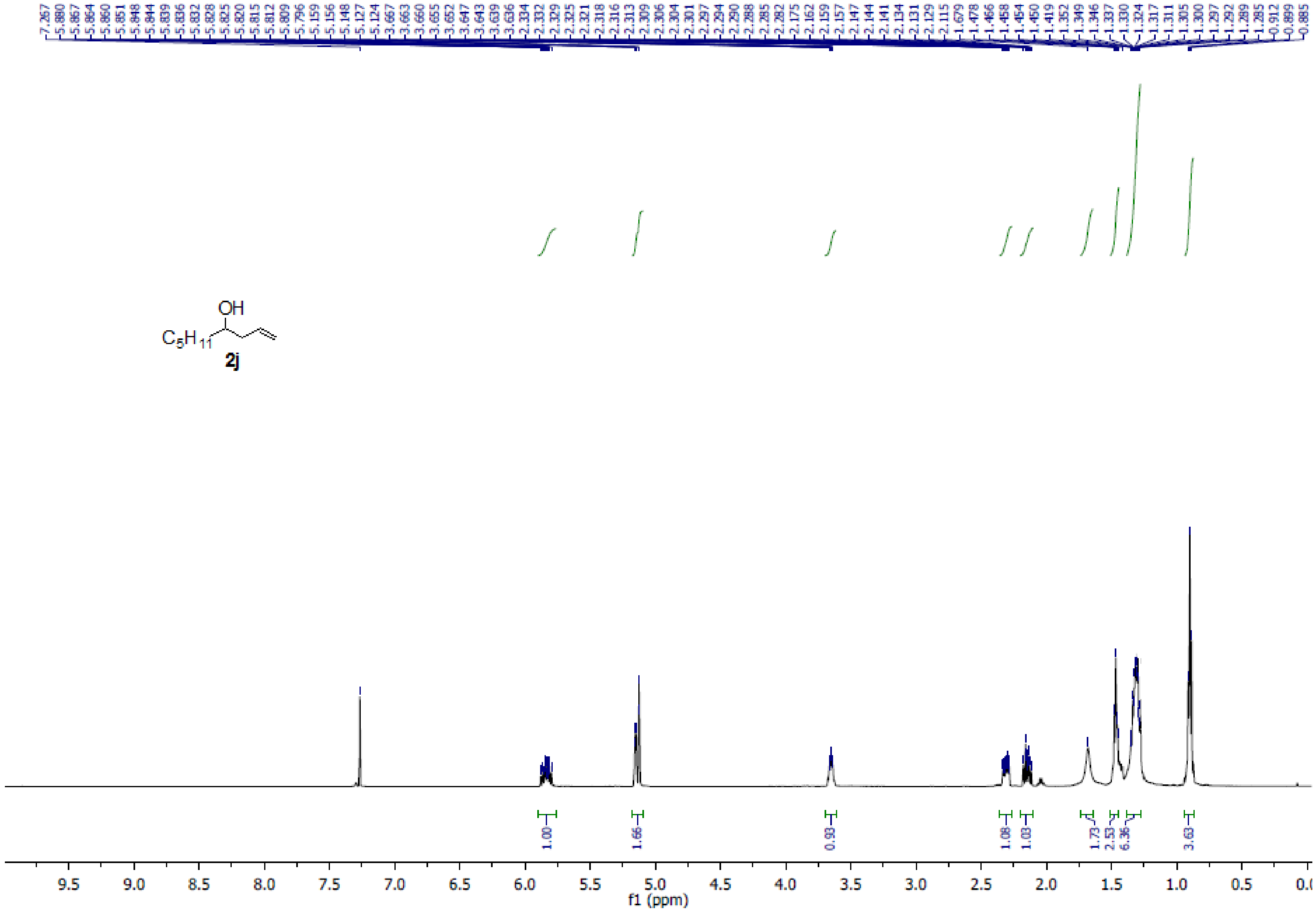

2j

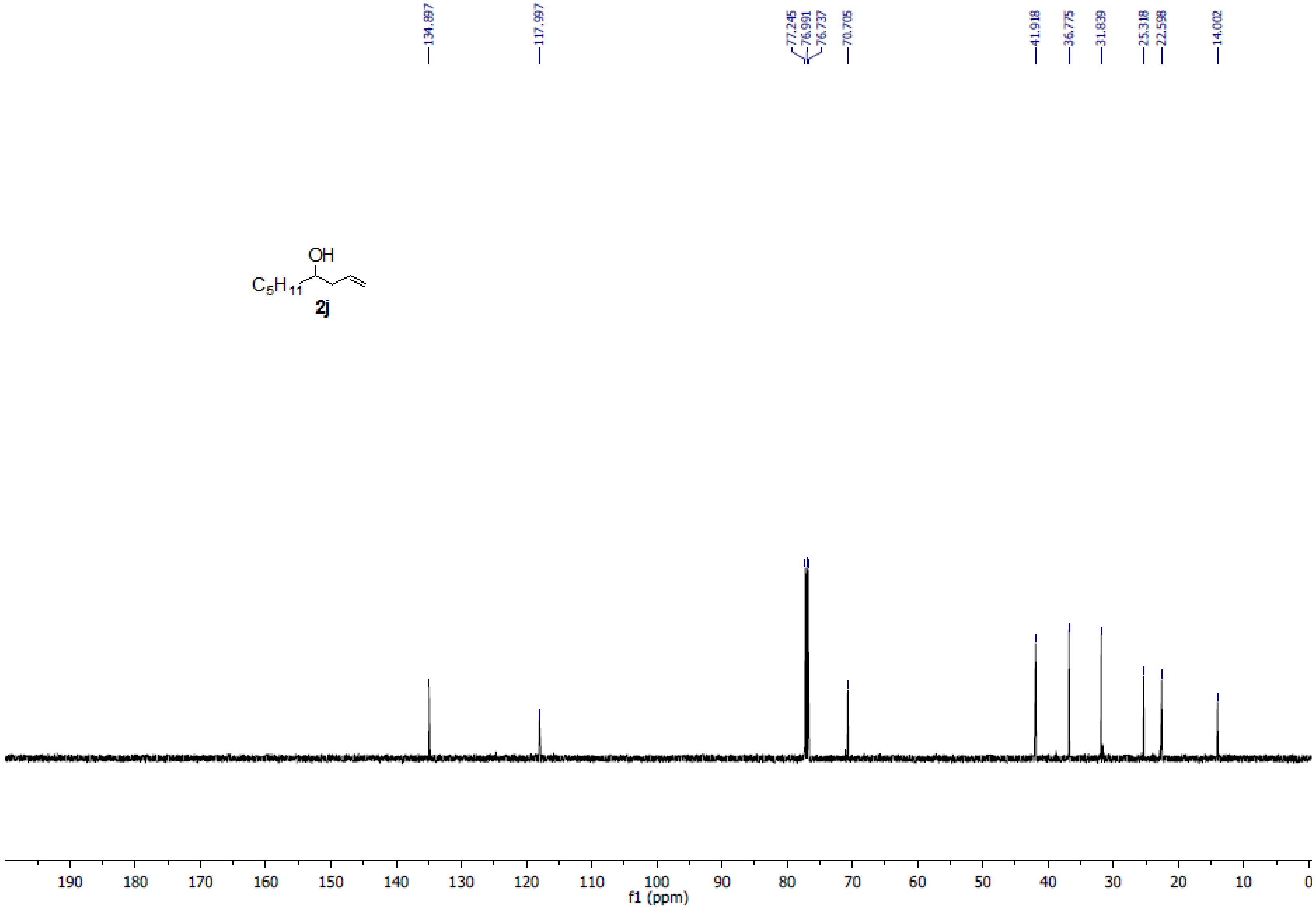
134.897
117.997
77.245
76.991
76.737
70.705
41.918
36.775
31.839
25.318
22.598
14.002
OH
C5H11
2j
190
180
170
160
150
140
130
120
110
100
90
80
70
60
50
40
30
20
10
0
f1 (ppm)

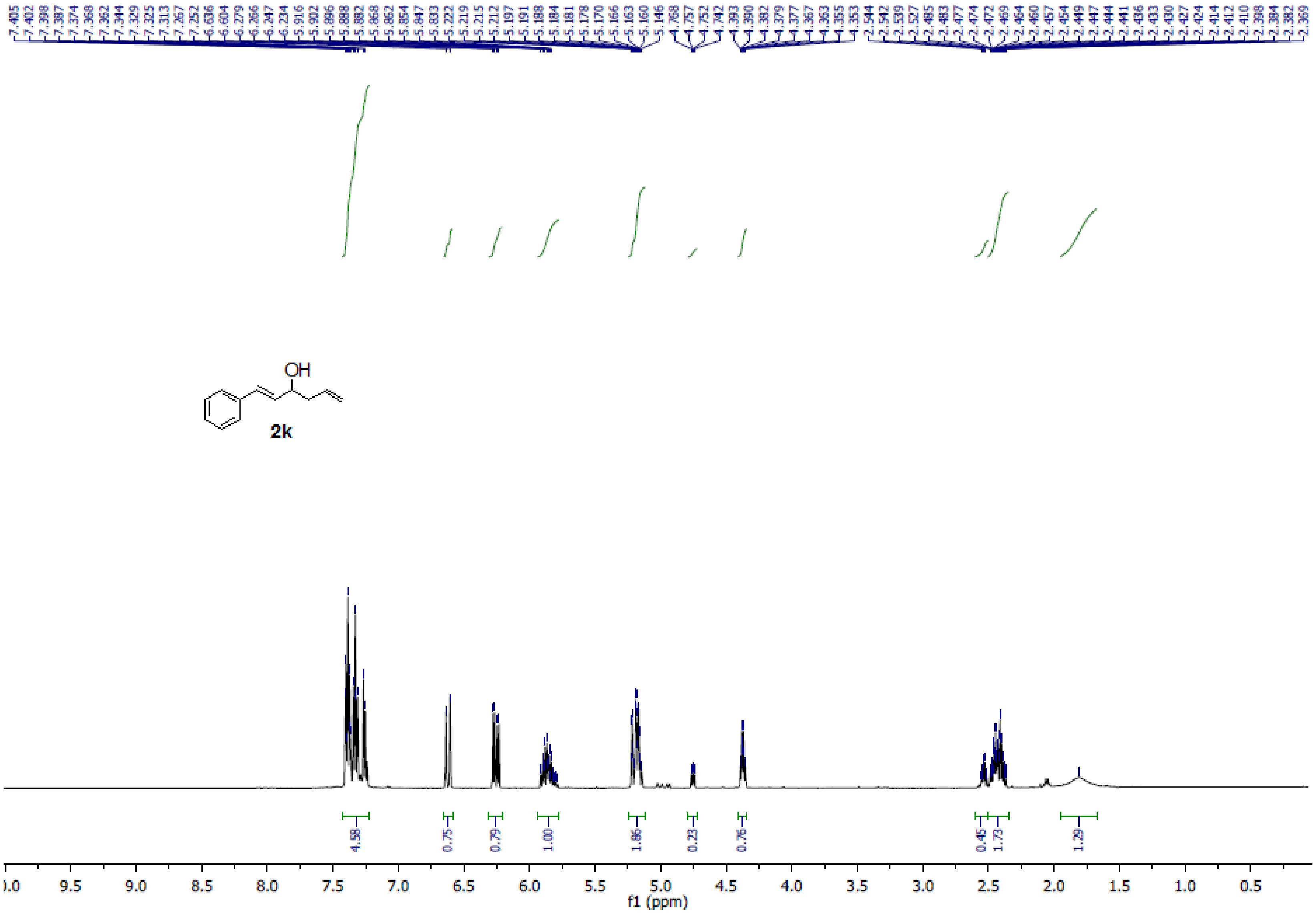
OH
2k
4.58
0.75
0.79
1.00
1.86
0.23
0.76
0.45
1.73
1.29
9.5
9.0
8.5
8.0
7.5
7.0
6.5
6.0
5.5
5.0
4.5
4.0
3.5
3.0
2.5
2.0
1.5
1.0
0.5
f1 (ppm)

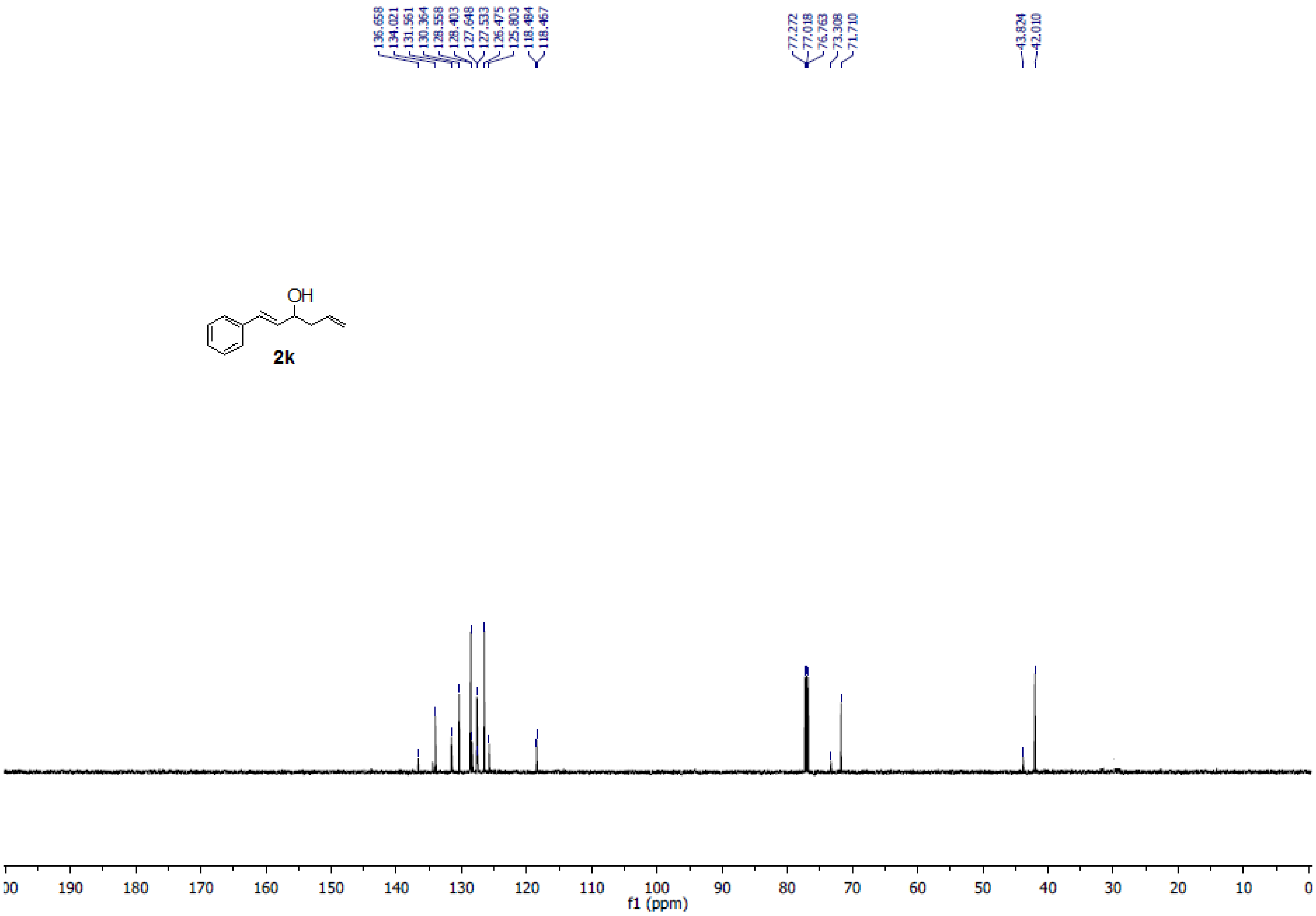
136.658
134.021
131.561
130.364
128.558
128.403
127.648
127.533
126.475
125.803
118.484
118.467
77.272
77.018
76.763
73.308
71.710
43.824
42.010
OH
2k
f1 (ppm)

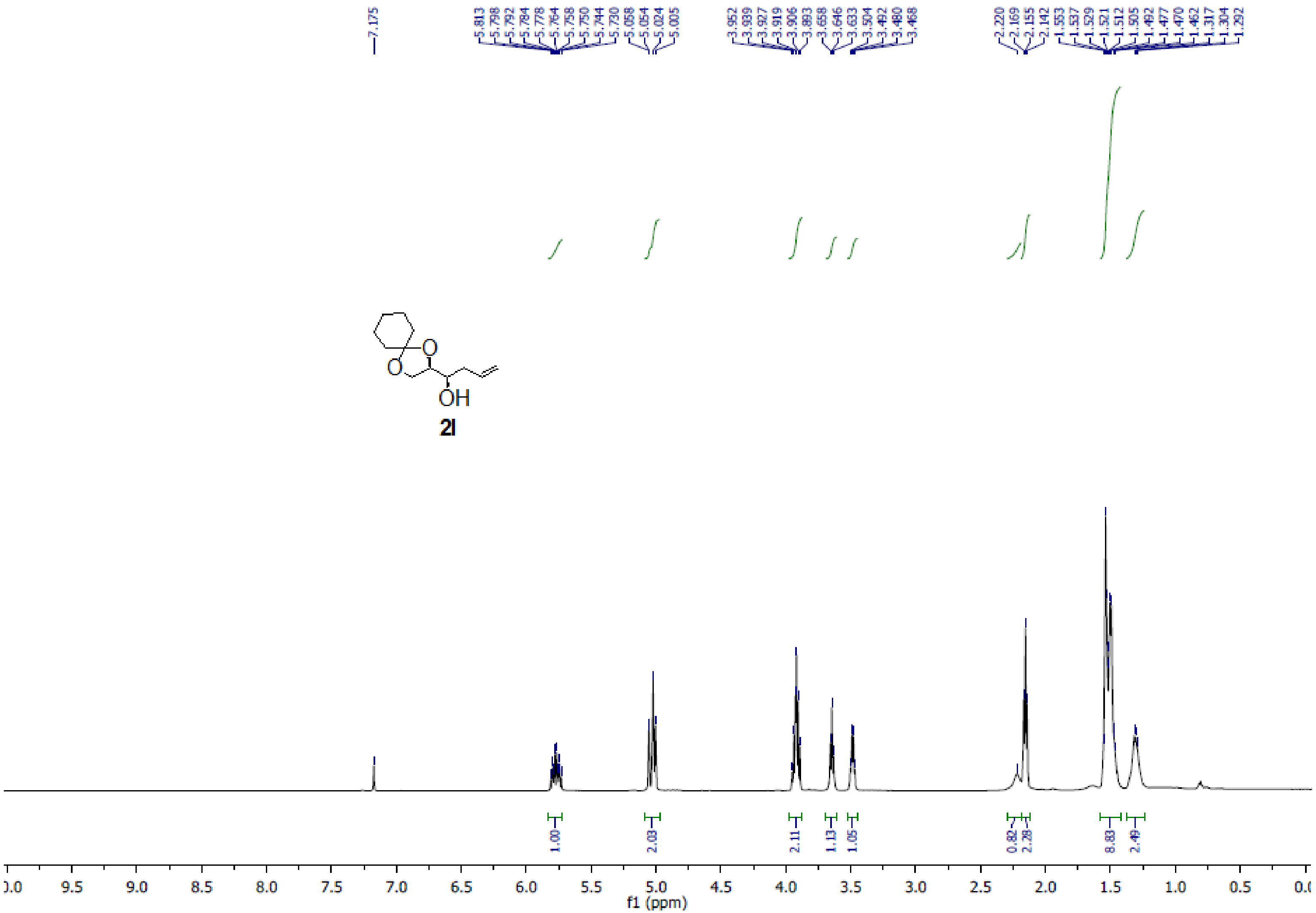

7.175
5.813 5.798 5.792 5.784 5.778 5.764 5.758 5.750 5.744 5.730 5.058 5.054 5.024 5.005
3.952 3.939 3.927 3.919 3.906 3.893 3.658 3.646 3.633 3.504 3.492 3.480 3.468
2.220 2.169 2.155 2.142 1.553 1.537 1.529 1.521 1.512 1.505 1.492 1.477 1.470 1.462 1.317 1.304 1.292
OH
2l
1.00
2.03
2.11
1.13
1.05
0.82
2.28
8.83
2.49
f1 (ppm)

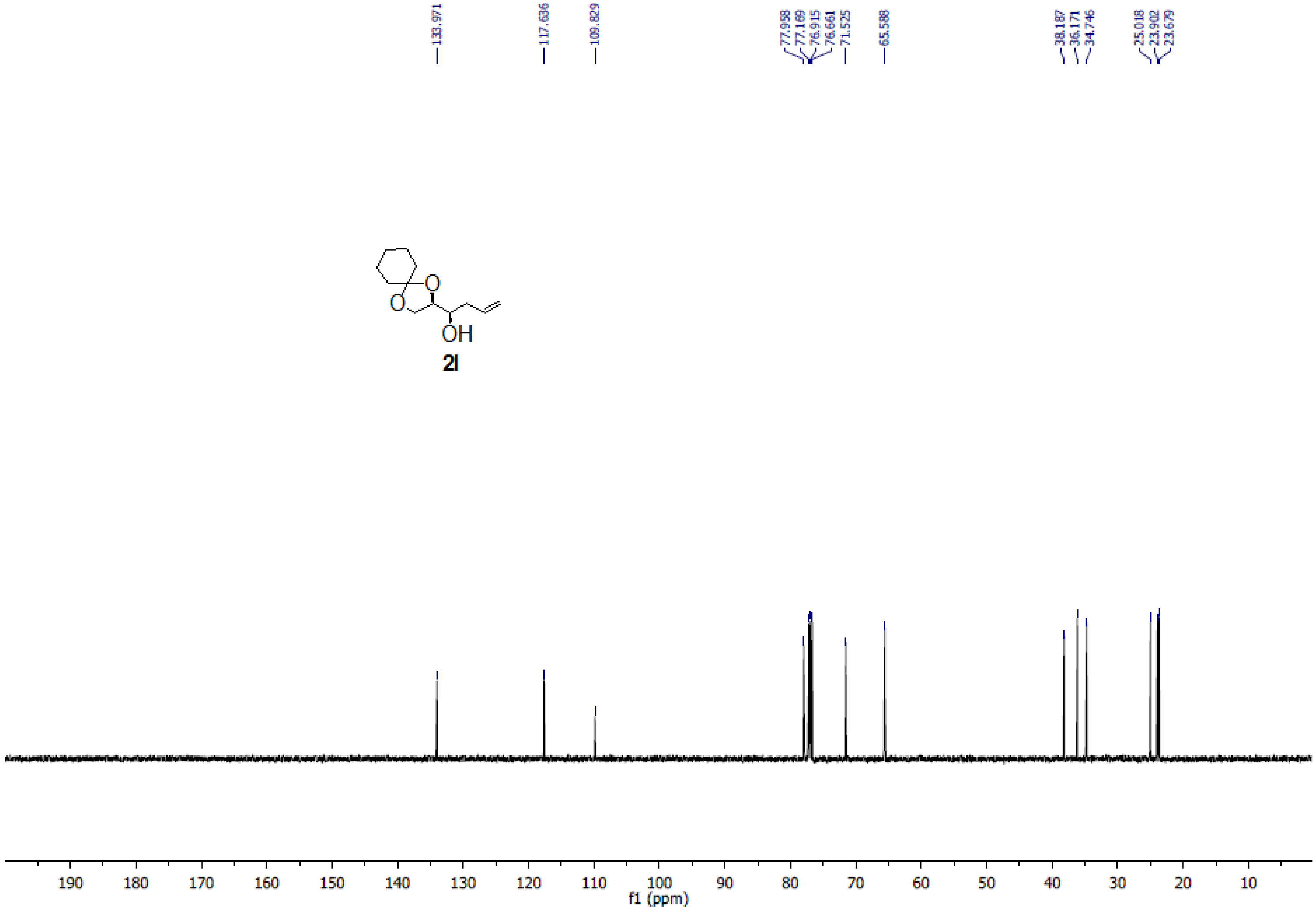
133.971
117.636
109.829
77.958
77.169
76.915
76.661
71.525
65.588
38.187
36.171
34.746
25.018
23.902
23.679
OH
2l
190
180
170
160
150
140
130
120
110
100
90
80
70
60
50
40
30
20
10
f1 (ppm)

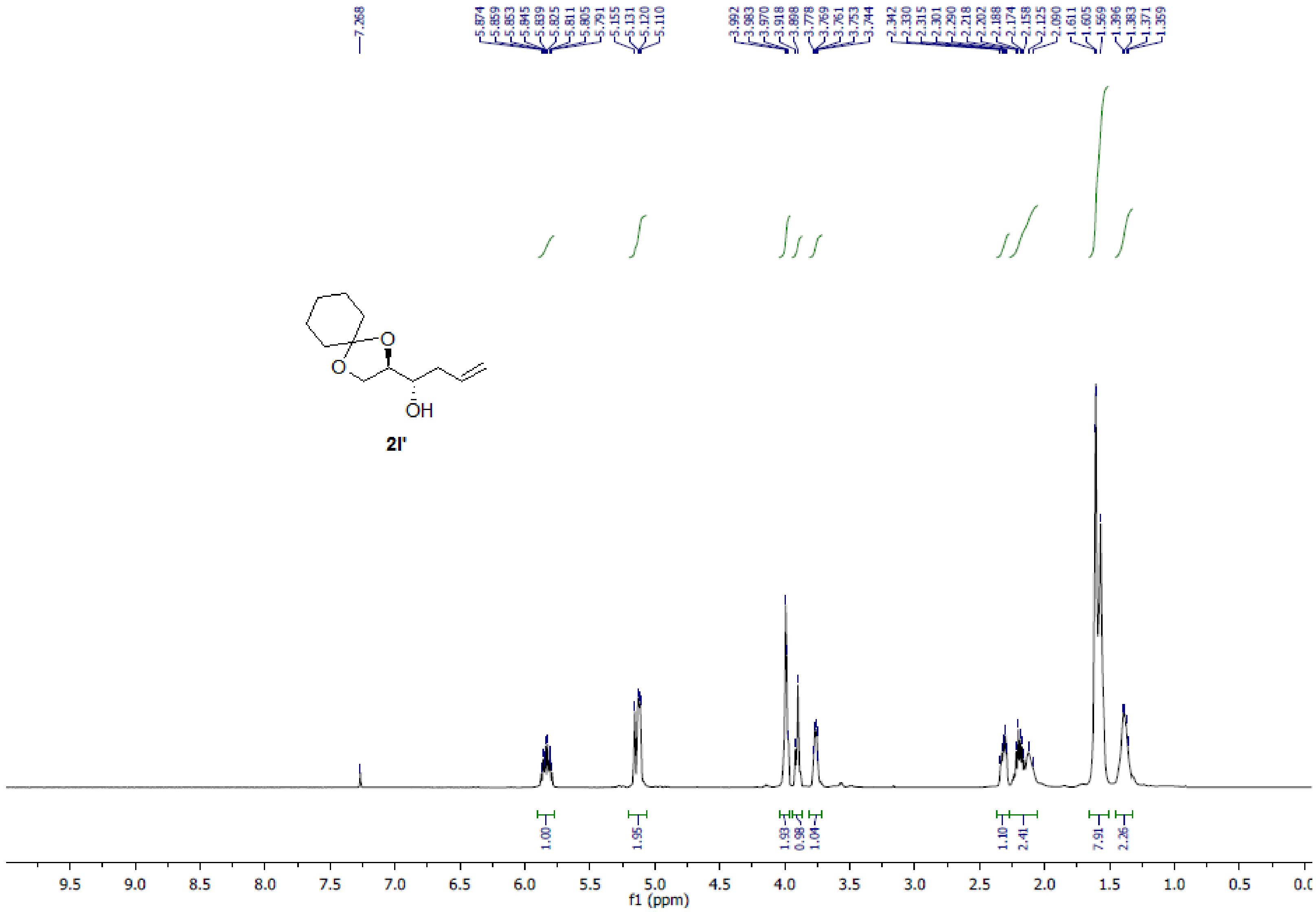
2l'
f1 (ppm)

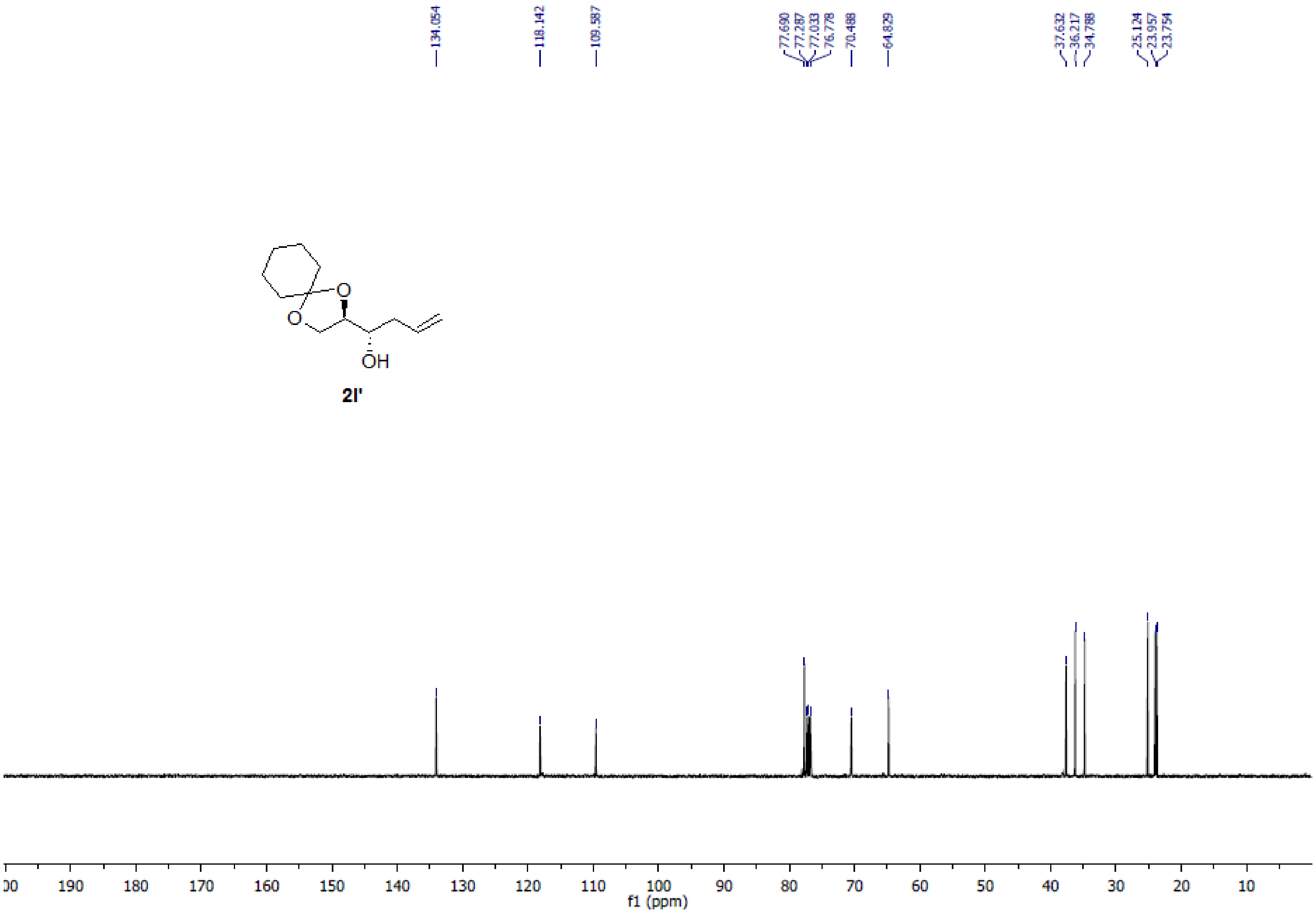
134.054
118.142
109.587
77.690
77.287
77.033
76.778
70.488
64.829
37.632
36.217
34.788
25.124
23.957
23.754
2l'
190
180
170
160
150
140
130
120
110
100
90
80
70
60
50
40
30
20
10
f1 (ppm)

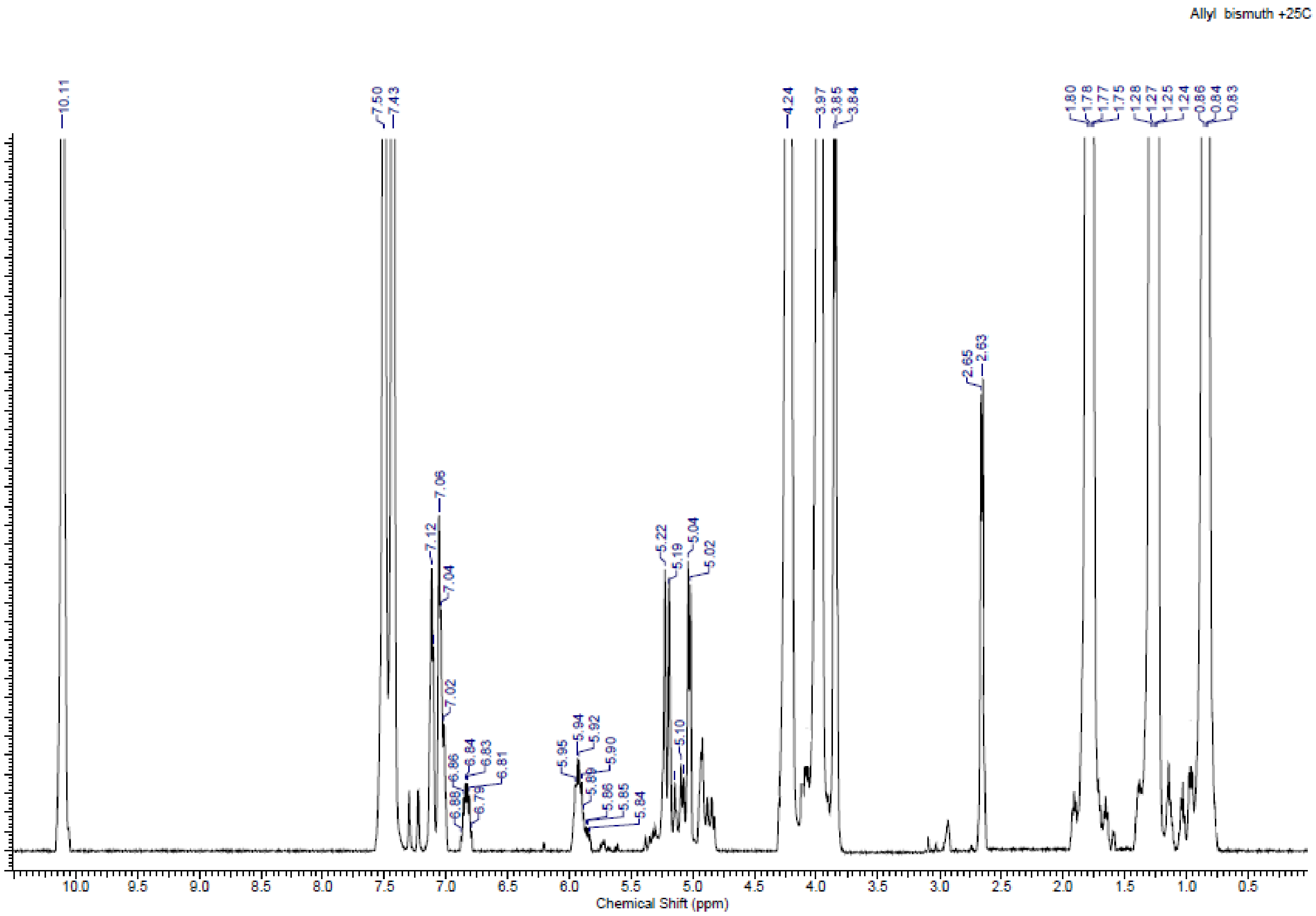

Figure S1:. Formation of **I**, as evident from ^{1}H NMR spectrum (recorded at room temperature in CD_2Cl_2) of a stirred mixture of Bi and allyl bromide in [bmim][Br].

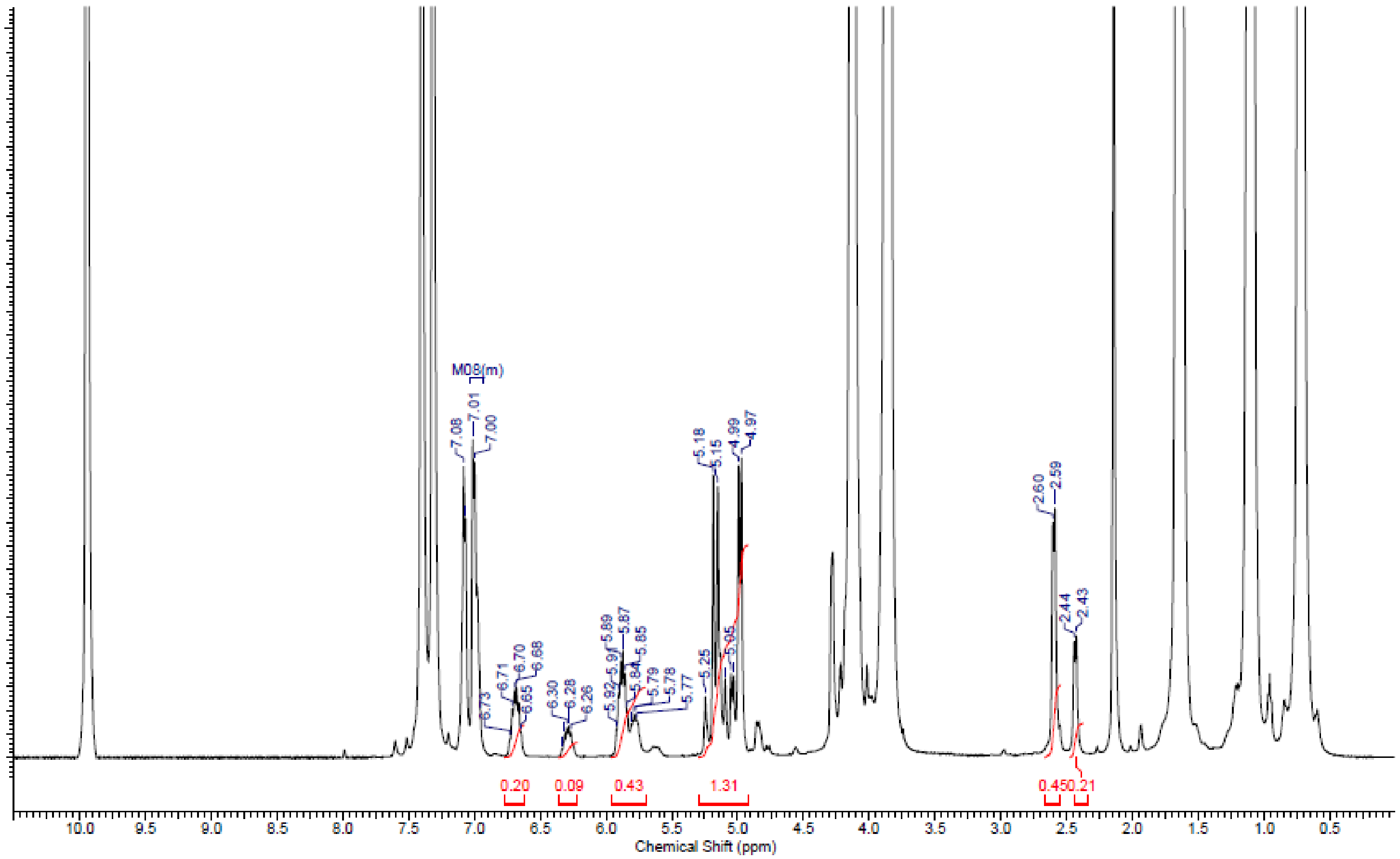

Figure S2: Formation of both **I** and **II**, as evident from ^{1}H NMR spectrum (recorded at -70 °C in CD_2Cl_2) of a stirred mixture of Bi and allyl bromide in [bmim][Br].
